# Strontium-incorporated hydroxyapatite nanocomposites promoting bone formation and angiogenesis by modulating M2 macrophage polarization in the bone microenvironment

**DOI:** 10.1093/rb/rbaf066

**Published:** 2025-06-23

**Authors:** Jing Li, Cuimiao Zhang, Jiayi Li, Ruijing Gao, Mengzhen Yang, Linkang Yu, Wei Zhang, Guoqiang Zhou, Wenzeng Shen, Jinchao Zhang, Guang Jia, Kun Ge

**Affiliations:** College of Chemistry & Materials Science, Key Laboratory of Medicinal Chemistry and Molecular Diagnosis of Ministry of Education, State Key Laboratory of New Pharmaceutical Preparations and Excipients, Chemical Biology Key Laboratory of Hebei Province, Hebei University, Baoding 071002, China; College of Chemistry & Materials Science, Key Laboratory of Medicinal Chemistry and Molecular Diagnosis of Ministry of Education, State Key Laboratory of New Pharmaceutical Preparations and Excipients, Chemical Biology Key Laboratory of Hebei Province, Hebei University, Baoding 071002, China; College of Chemistry & Materials Science, Key Laboratory of Medicinal Chemistry and Molecular Diagnosis of Ministry of Education, State Key Laboratory of New Pharmaceutical Preparations and Excipients, Chemical Biology Key Laboratory of Hebei Province, Hebei University, Baoding 071002, China; College of Chemistry & Materials Science, Key Laboratory of Medicinal Chemistry and Molecular Diagnosis of Ministry of Education, State Key Laboratory of New Pharmaceutical Preparations and Excipients, Chemical Biology Key Laboratory of Hebei Province, Hebei University, Baoding 071002, China; College of Chemistry & Materials Science, Key Laboratory of Medicinal Chemistry and Molecular Diagnosis of Ministry of Education, State Key Laboratory of New Pharmaceutical Preparations and Excipients, Chemical Biology Key Laboratory of Hebei Province, Hebei University, Baoding 071002, China; College of Chemistry & Materials Science, Key Laboratory of Medicinal Chemistry and Molecular Diagnosis of Ministry of Education, State Key Laboratory of New Pharmaceutical Preparations and Excipients, Chemical Biology Key Laboratory of Hebei Province, Hebei University, Baoding 071002, China; Institute of Biomedical and Health Engineering, Shenzhen Institute of Advanced Technology Chinese Academy of Sciences, Shenzhen 518055, China; College of Chemistry & Materials Science, Key Laboratory of Medicinal Chemistry and Molecular Diagnosis of Ministry of Education, State Key Laboratory of New Pharmaceutical Preparations and Excipients, Chemical Biology Key Laboratory of Hebei Province, Hebei University, Baoding 071002, China; College of Basic Medical Science, Hebei University, Baoding 071000, China; College of Basic Medical Science, Hebei University, Baoding 071000, China; College of Chemistry & Materials Science, Key Laboratory of Medicinal Chemistry and Molecular Diagnosis of Ministry of Education, State Key Laboratory of New Pharmaceutical Preparations and Excipients, Chemical Biology Key Laboratory of Hebei Province, Hebei University, Baoding 071002, China; College of Chemistry & Materials Science, Key Laboratory of Medicinal Chemistry and Molecular Diagnosis of Ministry of Education, State Key Laboratory of New Pharmaceutical Preparations and Excipients, Chemical Biology Key Laboratory of Hebei Province, Hebei University, Baoding 071002, China; College of Chemistry & Materials Science, Key Laboratory of Medicinal Chemistry and Molecular Diagnosis of Ministry of Education, State Key Laboratory of New Pharmaceutical Preparations and Excipients, Chemical Biology Key Laboratory of Hebei Province, Hebei University, Baoding 071002, China

**Keywords:** strontium-incorporated hydroxyapatite nanocomposites, effect of strontium concentration, bone formation, treatment of osteoporosis, bone injury microenvironment

## Abstract

The treatment of osteoporosis is urgently needed in the clinic. Hydroxyapatite (HAP) has a bone-inducing ability on osteogenic differentiation. Especially, the presence of strontium component in HAP nanoparticles may improve the positive effect on bone regeneration and avoid undesirable bone resorption. However, the incorporating concentrations of strontium still need to be elucidated to balance the osteogenic function and side effects. Herein, a series of strontium-incorporated HAP nanocomposites (Srx-HAP) with different Sr incorporating molar ratio concentrations (0%, 1%, 2%, 5%, 10%, 20%, 50%, 80% and 100%) have been prepared by a simple hydrothermal route. The Srx-HAP samples exhibited uniform and well-dispersed rod-like morphology, mesoporous structure, eminent degradability and good biocompatibility. In particular, Sr20-HAP exhibited prominent advantages in osteogenic differentiation and mineralization of pre-osteoblasts cell line MC3T3-E1. Sr20-HAP nanoparticles were highly effective in enhancing the bone formation in the rat model of postmenopausal osteoporosis compared to the ovariectomy group. In addition, Sr20-HAP nanoparticles could regulate macrophage polarization to M2 type *in vivo* and *in vitro*, providing an anti-inflammatory bone microenvironment and promoting bone repair and angiogenesis. This study provides a new insight of strontium-incorporated hydroxyapatite nanoparticles as competent anti-osteoporotic biomaterials for bone formation.

## Graphical abstract

**Figure:**
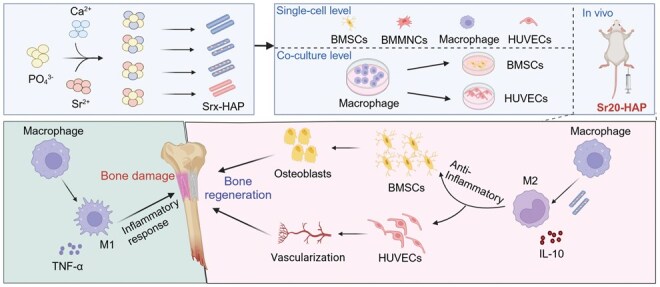

## Introduction

Osteoporosis that commonly occurs in the older can lead to fractures due to bone loss with impairment of bone microarchitecture and may aggravate with advancing age, which results in clinical burden and increased mortality [1, 2]. The pathophysiological mechanism of osteoporosis exhibits osteolysis caused by the disturbance of bone remodeling affected by bone resorption and new bone formation [1, 3]. There are two kinds of cells, osteoclasts (bone resorption) and osteoblasts (bone formation), involved and they have an interdependent relationship in bone remodeling [3]. The anti-resorptive treatments in clinics mainly comprise bisphosphonates, denosumab and selective estrogen receptor modulators [4]. Bisphosphonates, as the primary anti-osteoporotic agents, can inhibit osteoclast activity but are poor in bone remodeling because of their weak function in bone formation [5]. Long-term therapy with bisphosphonates, on the one hand, could show antifracture action, but on the other hand, it could increase the risk of a typical fracture. Denosumab can restrain bone resorption by binding to the receptor activator of the nuclear factor-κβ ligand (RANKL) to decline the osteoclasts differentiation. Compared with bisphosphonates, atypical femur fractures and osteonecrosis of the jaw have been rarely reported with denosumab treatment [6]. Estrogen treatment directly affects osteoblasts and osteoclasts, which can inhibit bone resorption and maintain bone formation. However, estrogen replacement is generally considered to be unfavorable due to concerns regarding the balance of risks and benefits in older women [4, 6].

To address the limitations of anticatabolic treatments for osteoporosis, new anabolic therapies aimed at stimulating osteoblasts and their precursors are being developed [6, 7]. However, only limited clinically approved treatments can increase bone formation. It is highly desirable to develop more potential candidate drugs for increasing osteogenesis. Numerous studies indicate HAP, the inorganic phase of the bone matrix, have a strong bone-inducing ability that promotes proliferation and osteogenic differentiation of osteoblastic lineage and progenitor cells [8–10]. HAP can contribute to tissue regeneration and be absorbent for biological substances and agents. Kaur *et al.* prepared the nanosized and microsized europium-doped HAP materials, which showed effectiveness in promoting bone formation in osteoporotic rats by intrafemoral injection [11]. Meanwhile, HAP crystals are also a good host ions matrix while keeping the same geometry. Strontium (Sr), as a normal trace element in the body, has a chemical analogy to Ca, which can accumulate in bone and positively affect bone formation at tissue and cellular levels [12, 13]. It was reported that the therapy on osteoporosis with Sr-incorporated HAP nanocomposites (Sr-HAP) exhibited enhancement in trabecular bone microarchitecture and mechanical strength compared to the treatment with pure HAP nanoparticles [14–17]. Moreover, bone resorptive serum biomarkers in the treatment with Sr-HAP were precluded compared to therapy with pure HAP nanoparticles [18, 19]. These findings not only indicate the potential of Sr-HAP nanocomposites in enhancing bone regeneration but also offer a promising future with reduced undesirable bone resorption in osteoporosis treatment.

However, the Sr content should be considered in the Sr-HAP materials because of its side effect [4, 20]. Sr displays a multiphasic and dose-dependent effect on bone formation *in vitro* and *in vivo*. In contrast, a low Sr dose administration led to bone formation, whereas a high Sr dose induced disturbed mineralization [21]. Although some studies reported that Sr-HAP nanocomposites positively affected the osteoporosis rat [12], the incorporating concentrations of Sr in HAP still need to be elucidated to balance the osteogenic function and side effects. Moreover, the bone microenvironment contains osteoblasts, osteoclasts, immunocytes and vascular endothelial cells. There are close interactions among various cells, and studying a single cell makes it difficult to comprehensively understand the mechanism of HAP on bone formation, therefore, the mechanism of Sr-HAP regulating bone formation still needs to be examined, highlighting the need for further research in this area.

Herein, we developed a series of Srx-HAP nanocomposites with different Sr incorporating molar ratio concentrations (0%, 1%, 2%, 5%, 10%, 20%, 50%, 80% and 100%) by a facile hydrothermal method, and investigated their physicochemical and morphological properties. Moreover, we systematically studied the preosteoblasts’ behavior in the proliferation and differentiation in the light of the biomaterial compositional changes. Furthermore, we studied the mechanism of Srx-HAP on the bone formation in the single cellular behavior and interactions of osteoblasts, osteoclasts, macrophages and vascular endothelial cells and the phenotype of bone microenvironment-related cells in the old-aged mice. This work aims to provide insights into the potential of Sr-incorporated HAP nanocomposites as competent anti-osteoporotic biomaterials for bone formation, underscoring the significance of our research.

## Materials and methods

### Materials and reagents

Cetyl trimethyl ammonium bromide (CTAB, 99%), 3-(4,5-dimethylthiazol-2-yl)-2,5-diphenyltetrazolium bromide (MTT), β-glycerophosphate, ascorbic acid, dexamethasone and macrophage colony-stimulating factor (M-CSF) were obtained from Sigma-Aldrich. Alendronate (ALN) was purchased from Dalian Meilun Biotech Co. Ltd. Trypsin was purchased from AMRESCO. Dulbecco’s modified eagle’s medium (DMEM), alpha-minimum essential medium (α-MEM), fetal bovine serum (FBS) and penicillin-streptomycin were purchased from ThermoFisher. Alkaline phosphatase (ALP) activity detection kit, BCA assay kit and ALP qualitative kit were obtained from Nan Jing Jian Cheng Bioengineering Inc. Rat CTX-1 (carboxy-terminal telopeptides of type I collagen) enzyme immunoassay kit was purchased from Cloud-Clone Corporation (Wuhan, China). CCK-8 kit was obtained from Biyuntian Biotechnology Company. RANKL was obtained from R&D Biotechnology Company. Alizarin red staining reagent was purchased from Beijing Solarbio Technology Company. Arg-1 Antibody, iNOS Antibody, CD31 antibody and CD68 antibody were purchased from Abcam. Other reagents were all analytical grade and were used directly.

### Cells lines and animal feeding

Human umbilical vein endothelial cells (HUVECs), pre-osteoblasts cell line MC3T3-E1 and Raw264.7 cells were sourced from the National Infrastructure of Cell Line Resource. Bone marrow-derived mesenchymal stem cells (BMSCs) were isolated from the bone marrow of the limbs of 4-week-old ICR mice. Bone marrow mononuclear cells (BMMNCs) were harvested from the bone marrow of the limbs of 9-week-old ICR mice. 4-week-old and 9-week-old ICR mice, 12-week-old female BALB/c mice and 12-week female Wistar rats, were provided from Beijing Vital River Laboratory Animal Technology Co., Ltd. All animal experiments were carried out at the Medical Comprehensive Experimental Center of Hebei University according to the rules of the Animal Welfare and Ethical Committee of Hebei University (approval number IACUC-2018025).

### Preparation and characterization of Srx-HAP nanocomposites

The pure HAP nanoparticles were fabricated based on our previous work [22], which was described in detail in the Supplementary Material. A similar synthesis procedure was employed to fabricate the Srx-HAP nanocomposites except for introducing a stoichiometric amount of Sr(NO_3_)_2_ instead of Ca(NO_3_)_2_ at the initial step [23]. The designed Sr^2+^/(Ca^2+^+Sr^2+^) molar ratios of 0.01, 0.02, 0.05, 0.1, 0.2, 0.5, 0.8 and 1 for the Srx-HAP samples were labeled as Sr1-HAP, Sr2-HAP, Sr5-HAP, Sr10-HAP, Sr20-HAP, Sr50-HAP, Sr80-HAP and Sr100-HAP (pure SrHAP), respectively. The characterizations of the Srx-HAP nanocomposites were ascertained by X-ray diffraction (XRD) patterns, scanning electron microscope (SEM), transmission electron microscopy (TEM) and nitrogen adsorption/desorption analysis [24–28]. The detailed information was listed in the Supplementary Material.

### Biosafety and bone formation evaluation of Srx-HAP

Pre-osteoblasts cell line MC3T3-E1 was first employed for the cellular safety determination of nine kinds of Srx-HAP by MTT method at 24, 48 and 72 h. Thereafter, the ovariectomy osteoporotic rats were administered with HAP, Sr10-HAP, Sr20-HAP and Sr100-HAP. The bone formation capability was carried out by micro-CT and serum bone resorption marker CTX-1 [29]. Meanwhile, the blood and organs safety were determined by the blood-element test [30], biochemical parameters in the serum and hematoxylin and eosin (H&E) staining. The detailed information was listed in the Supplementary Material.

### Cell viability and functional assay of BMSCs, BMMNCs, HUVECs and Raw264.7 cells treated with HAP and Sr20-HAP

We assessed the cell viability of BMSCs, BMMNCs, HUVECs and Raw264.7 cells on HAP and Sr20-HAP was assessed in this study, considering their significance in the bone microenvironment. Furthermore, the osteogenesis of BMSCs, osteoclast differentiation of BMMNCs, vascularization of HUVECs and macrophage phenotype of Raw264.7 cells under the treatment of HAP and Sr20-HAP were evaluated by alkaline phosphatase (ALP) staining, ALP activity and mineralization analysis, tartrate resistant acid phosphatase (TRAP) staining, scratch test, tube formation assay and western blot for M1 and M2 macrophage phenotype based on our previous work [31]. The detailed information was listed in the Supplementary Material.

### Co-culture of Raw264.7 cells with BMSCs or HUVECs

To investigate the impact of different macrophage phenotypes of Raw264.7 on the osteogenic differentiation capacity of BMSCs and the angiogenic ability of endothelial cells, we co-cultured nanomaterial-incubated medium with Raw264.7 cells and separately cultured BMSCs and HUVECs [32, 33]. The purpose of this experiment was to simulate the *in vivo* environment where different cell types interact and influence each other’s functions. Initially, HAP and Sr20-HAP at a concentration of 1 μg/mL were incubated with Raw264.7 cells for 6 and 72 h, and a conditioned medium was prepared by mixing their culture supernatant with a fresh complete medium at a ratio of 1:10 for culturing BMSCs and HUVECs. Subsequently, we evaluated the osteogenic differentiation and mineralization capacity of BMSCs and the migration and angiogenesis ability of HUVECs.

### Old-aged mice model experiments

Considering that bone health deteriorates with age, understanding the effects of Sr20-HAP in an aging bone microenvironment is crucial for potential clinical applications [34]. We utilized old-aged female BALB/c mice to investigate the impact of Sr20-HAP on bone formation by regulating the bone microenvironment *in vivo*. Eighteen 12-month-old mice were randomly divided into three groups, each receiving an intratibial injection of saline, HAP or Sr20-HAP (at a dosage of 30 mg/kg), respectively. Concurrently, six 4-week-old mice were included and assigned as a control group. After 2 weeks, mice were anesthetized with isoflurane to analyze the bone immune microenvironment. After 4 weeks, the mice were killed by eyeball blood collection. The biosafety of HAP and Sr20-HAP *in vivo* was conducted by blood biochemistry and mainly tissue organs H&E analysis. The trabecular structures of the tibia distal region were characterized by micro-CT and morphological observation [35].

### The bone immune microenvironment analysis *in vivo*

After fixation of the mice’s tibia in a 4% paraformaldehyde solution for 24 h, the leg bones were rinsed with ultra-pure water and immersed in a 10% EDTA (pH 8.0) solution for decalcification at room temperature. After decalcification, the samples underwent dehydration in gradient ethanol and embedding in paraffin wax. Subsequently, bone tissue sections with a thickness of 5 μm were obtained using a Leica microtome. H&E staining was performed along with immunofluorescence staining for CD68, iNOS and Arg-1 to identify macrophage phenotypes. Additionally, immunohistochemical staining was conducted to detect IL-10, TNF-α and CD31 antibodies within bone slices. All stained sections were observed and documented under a microscope.

### Statistical analysis

Experiments were carried out in triplicate, and the outcomes were presented in the form of mean ± standard deviation. For the statistical evaluation between two groups, the t-test was applied, whereas one-way analysis of variance (ANOVA) was resorted to when it came to the comparison involving three or more groups.

## Results and discussion

### Crystal structure, morphology and luminescence properties

HAP, as the predominant inorganic constituent of human bone tissue, has gained significant attention in biomedical engineering due to its exceptional physicochemical properties, particularly in targeted drug delivery systems, bioimaging applications and bone regeneration therapies [36]. Notably, Sr and Ca, belonging to the same group of divalent alkaline earth metals, serve as essential trace elements in human physiological processes. Substantial experimental evidence indicates that Sr exhibits remarkable chemical and structural analogies with Ca, demonstrating inherent biocompatibility, negligible immunogenicity and efficient cellular uptake through both specific ion channels and passive membrane diffusion mechanism [37]. Importantly, Sr displays dual regulatory effects on bone remodeling by simultaneously enhancing osteoblast proliferation/differentiation and suppressing osteoclast-mediated bone resorption. Capitalizing on these unique characteristics, we successfully developed a series of Sr-incorporated HAP nanoparticles through controlled synthesis and systematically investigated their structure–property relationships for potential biomedical implementations.

**Figure 1.**
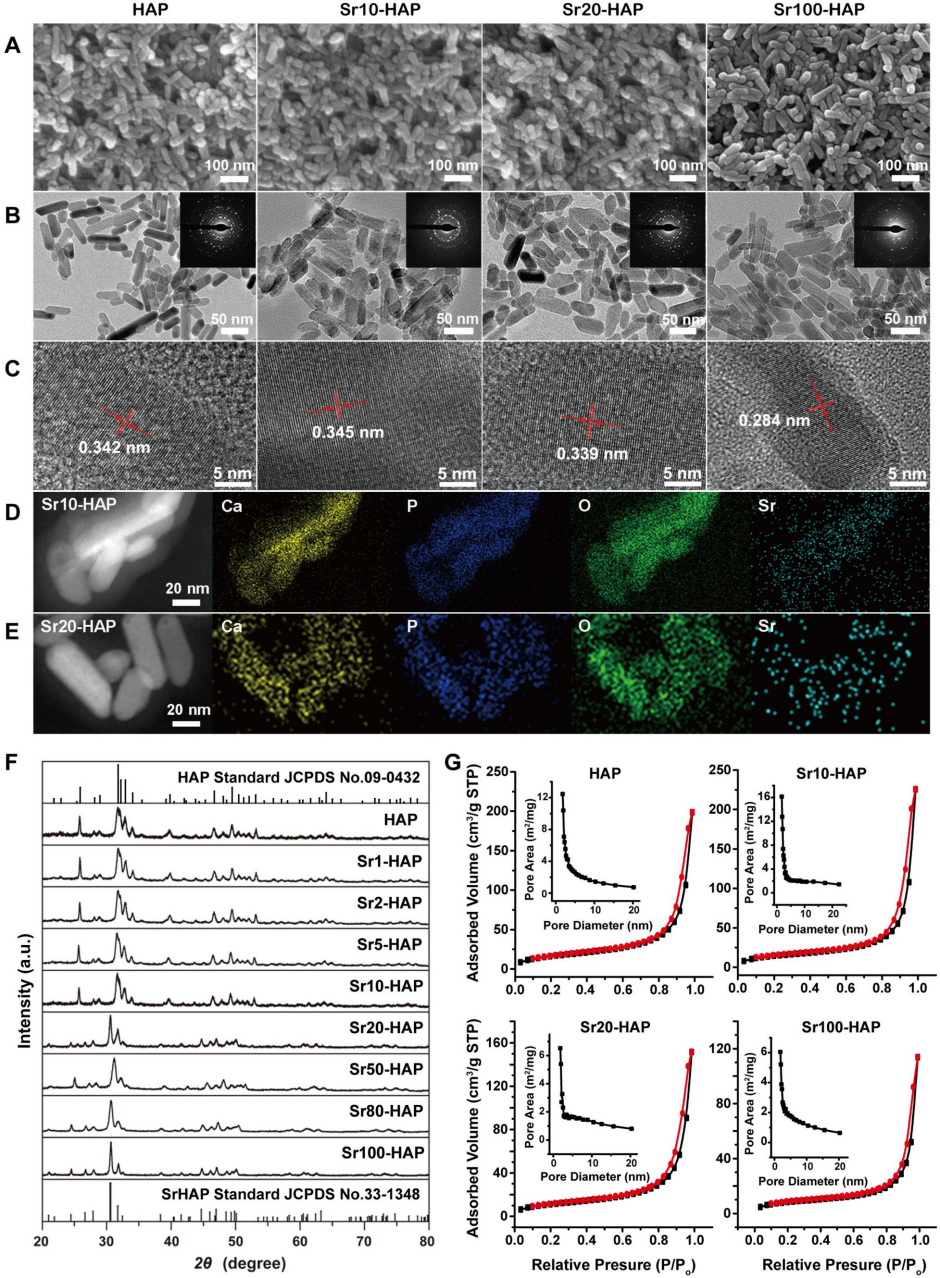
Characterizations of Srx-HAP nanocomposites. (**A**) SEM, (**B**) TEM and SAED patterns (insets) and (**C**) HRTEM images of Srx-HAP. EDX elemental mapping images of (**D**) Sr10-HAP and (**E**) Sr20-HAP. (**F**) XRD patterns of the Srx-HAP samples. (**G**) N_2_ adsorption/desorption isotherms and the corresponding BJH pore size distribution curves (insets) of Srx-HAP samples.

The as-synthesized pure HAP nanoparticles and Srx-HAP nanocomposites with different proportions of Sr were prepared *via* a hydrothermal process. The SEM and TEM images showed that all the as-synthesized samples (HAP, Sr1-HAP, Sr2-HAP, Sr5-HAP, Sr10-HAP, Sr20-HAP, Sr50-HAP, Sr80-HAP and Sr100-HAP) were composed of uniform and well-dispersed rod-like particles with the lengths of 50 ∼80 nm and diameters of about 20 nm (Figure 1A and B, and Supplementary Figures S1 and S2). The SAED patterns (inset in Figure 1B) showed the regular dot ring patterns, revealing the polycrystalline structure of the samples. The distinct lattice fringes detected from the HRTEM revealed the high crystallinity of the Srx-HAP samples (Figure 1C). The interplanar distances between the adjacent lattice fringes were determined to be 0.342, 0.345 and 0.339 nm for pure HAP, Sr10-HAP, Sr20-HAP, which correspond to the (002) crystal planes of hexagonal HAP [38]. However, the distance of the Sr100-HAP sample was 0.284 nm, which agrees well with the d_300_ value of the hexagonal phase of Sr100-HAP. The result demonstrated that the phase structure of the Srx-HAP samples with low Sr incorporating concentrations belonged to the hexagonal phase of HAP, which was supported by the following XRD patterns. The elemental mapping images of Sr10-HAP and Sr20-HAP displayed the uniform dispersity of Ca, Sr, P and O elements (Figure 1D and E), which illustrated that the Sr element was homogeneously incorporated into the HAP host matrix. The actual incorporating concentrations of strontium in the HAP lattice were confirmed by ICP-OES (Supplementary Table S2). The actual incorporating concentrations of strontium were 1.225, 2.353, 6.264, 12.339, 22.797, 54.277, 80.443, 99.566 for Sr1-HAP, Sr2-HAP, Sr5-HAP, Sr10-HAP, Sr20-HAP Sr50-HAP, Sr80-HAP and Sr100-HAP samples, respectively. The actual incorporating concentrations were basically consistent with the feeding ratios, indicating the Sr^2+^ could be quantitatively incorporated into the HAP matrix by simply adjusting the relative feeding ratios of Ca(NO_3_)_2_ and Sr(NO_3_)_2_ at the initial synthesis step.

The XRD patterns of pure HAP and all Srx-HAP samples were shown in Figure 1F. The diffraction peaks of samples with low incorporating concentrations [Sr^2+^/(Ca^2+^ + Sr^2+^) ≤ 0.1] correspond to the hexagonal phase of HAP (JCPDS No. 09-0432). When the incorporating concentration increased to 0.2 (Sr20-HAP), the as-obtained Sr20-HAP sample exhibited the mixing phase of hexagonal HAP and Srx-HAP (JCPDS No. 33-1348), which was in accordance with the HRTEM result (Figure 1C). By further enhancing the incorporating concentrations to 0.5 (Sr50-HAP), 0.8 (Sr80-HAP) and 1.0 (Sr100-HAP), the crystal structure preferentially corresponded to the hexagonal phase of Srx-HAP. No peak shift and other calcium phosphate phases were detected from XRD patterns. The result revealed the crystal phase conversion from HAP to Srx-HAP by increasing the Sr incorporating concentrations. The respective N_2_ adsorption/desorption isotherms indicated that the HAP, Sr10-HAP, Sr20-HAP and Sr100-HAP samples displayed similar VI isotherms in IUPAC classification and the H1-hysteresis loops, revealing the mesoporous structure of the materials (Figure 1G). The hysteresis between the adsorption and desorption curves was because the adsorption and desorption processes were irreversible [39]. The inset in Figure 1G exhibited that the pore sizes of all the samples were about 3–5 nm. Moreover, the pure HAP sample showed the highest specific surface area (60.38 m^2^/g) and pore volume (0.31 cm^3^/g), and the surface area and pore volume decreased gradually by elevating the Sr^2+^ incorporating concentration (Supplementary Table S2). It could be summarized that the morphology, mesoporous structure and crystallinity of the samples were similar to the natural HAP in bone tissue, which would be favorable in bone tissue engineering [38].

### MC3T3-E1 cell viability and osteogenic differentiation capability *in vivo*

**Figure 2.**
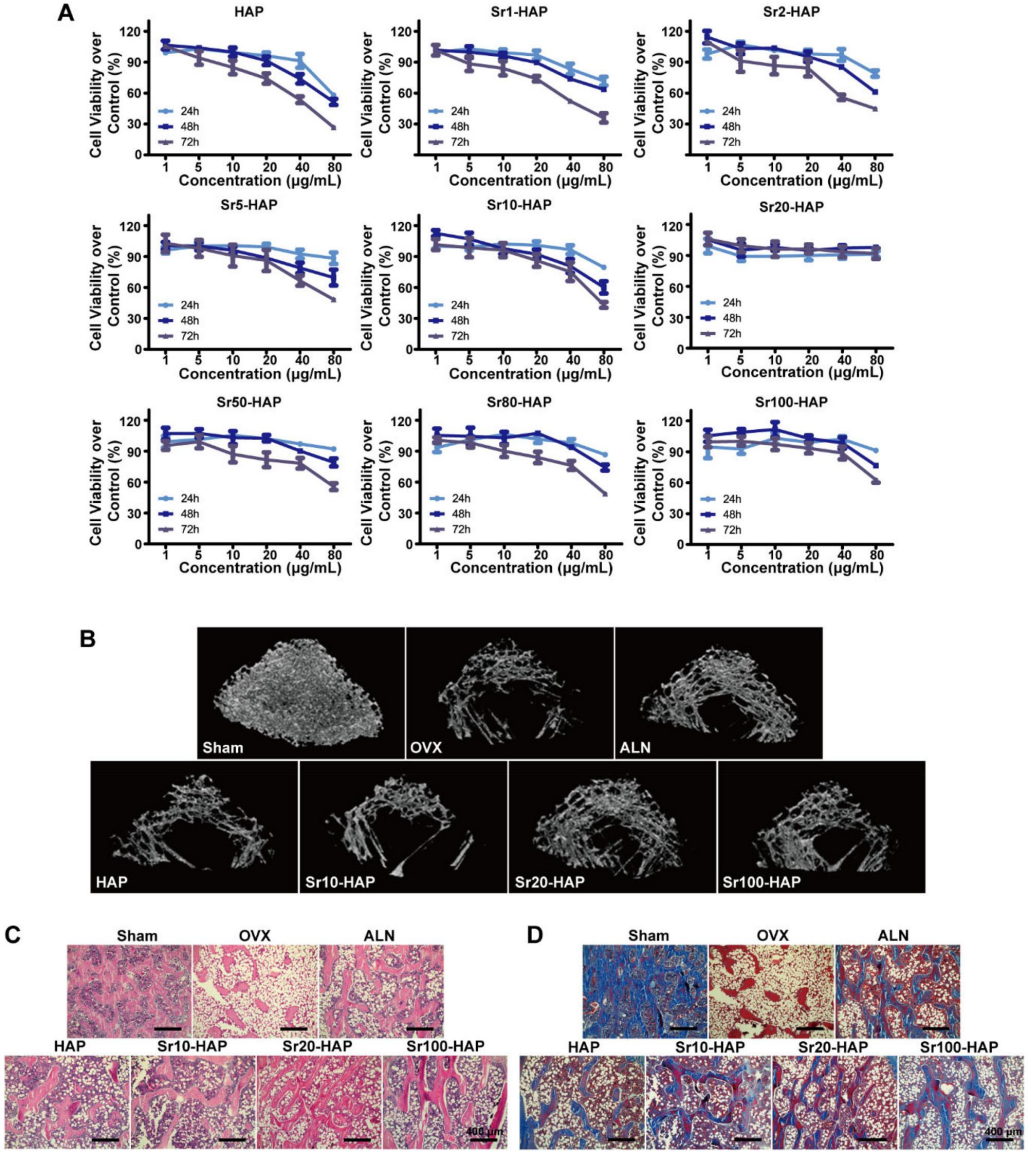
Cell viability and bone formation capability of osteoporosis rat after being treated with Srx-HAP *in vivo*. (**A**) Cell viabilities of MC3T3-E1 after incubation with the samples at different concentrations. (**B**) Micro-CT topview images of trabecular structure of distal femur. Micrographs of (**C**) H&E-stained and (**D**) Masson-stained femur trabecular.

Sr can promote the proliferation, differentiation and mineralization of osteoblasts [40]. When hydroxyapatite is doped with strontium, it can offer a more favorable microenvironment for the attachment, growth and differentiation of osteoblasts, thereby accelerating the formation of new bone tissue [41]. To identify the optimal Sr doping ratio that enhances the biological performance of HAP while avoiding potential cytotoxicity, the cell viabilities of Srx-HAP were first evaluated by MTT assay in pre-osteoblastic cell lines MC3T3-E1. Cell viabilities were investigated for 24, 48 and 72 h after incubation with Srx-HAP at different concentrations, respectively. Except for Sr20-HAP, the cell viabilities of MC3T3-E1 were inhibited in a dose-dependent manner when treated with all other Srx-HAPs at three-time points (Figure 2A). When the Sr content was less than 20%, the cell viabilities showed increasing tendency with the increase of Sr contents at 72 h. However, when the Sr content was more than 20%, the cell viabilities exhibited no obvious change. The differentiation of MC3T3-E1 treated with HAP, Sr10-HAP, Sr20-HAP and Sr100-HAP samples was monitored by ALP activity, which was a mid-stage biomarker for osteogenic differentiation [42–44]. Positive ALP production was significant upregulation at 14 days. HAP, Sr10-HAP, Sr20-HAP and Sr100-HAP samples display significantly higher ALP secretion levels than the OS control group, and Sr20-HAP showed the highest ALP level (Supplementary Figure S3A). Participating in biological mineralization is an important function of osteogenic cells. Therefore, calcium deposition by MC3T3-E1 cells was evaluated by alizarin red S staining and semiquantificational alizarin red-based assays. After incubation with Srx-HAP and MC3T3-E1 cells for 20 days, calcium secretion could be significantly observed from cells cultured by HAP, Sr10-HAP, Sr20-HAP and Sr100-HAP (Supplementary Figure S3B and C). Sr20-HAP and Sr100-HAP samples exhibited more mineralization nodules.

We next evaluated the bone formation effects of HAP, Sr10-HAP, Sr20-HAP and Sr100-HAP samples in osteoporotic rats by single intravenous injection (Supplementary Figure S4A). When the rats get osteoporosis, the body weight would change higher because of the estrogen deprivation. At the end of the animal experiment (rats’ age is about 36 weeks), the rats' weights in OVX, HAP and Sr100-HAP groups showed significant differences with the Sham group, but rats' weights in Sr10-HAP and Sr20-HAP groups exhibited no significant difference with that of Sham group (Supplementary Figure S4B). And the main organs of all groups seemed no significant changes (Supplementary Figure S4C). Meanwhile, there were little changes on the biochemical parameters in the serum (Supplementary Table S3) and blood-element (Supplementary Table S4) in Srx-HAP groups. As could be seen from the trabecular structure of the distal femur detected by micro-CT, it was found that OVX, HAP, Sr10-HAP, Sr20-HAP and Sr100-HAP groups all displayed significant bone loss compared with the Sham group (Figure 2B). The trabecular volume and number showed the increase in Sr20-HAP’s group in contrast with the OVX group (Supplementary Figure S5A and B). However, the trabecular structure in other three Srx-HAP groups showed little change compared to the OVX group (Figure 2C and D). Ovariectomy induced significant osteolysis in estrogen-deficient rats. The serum CTX-1 contents in the OVX group were significantly higher than that of Sham group (Supplementary Figure S5C). Based on these results, Sr20-HAP demonstrated superior biosafety and osteogenic capacity *in vitro* and *in vivo*. Therefore, it was selected for further mechanistic investigations.

### Cell viability and biological effects of HAP and Sr20-HAP for the bone microenvironment-related cells

The biological effects of Sr ions in bone metabolism involve complex mechanisms on various cells in the bone microenvironment. The cell viability of HAP and Sr20-HAP was thoroughly evaluated for their potential use in bone-related research. Supplementary Figure S6A and B demonstrated an excellent safety profile and showed that HAP and Sr20-HAP had good safety against BMSCs cells at 1 and 5 μg/mL. However, it was important to note that the cell activity gradually decreased with increased incubation concentration and time. HAP and Sr20-HAP had a strong inhibitory effect on the cell viability of BMMNC, and with the rise of HAP and Sr20-HAP concentration and time, the cell viability gradually decreased (Supplementary Figure S6C and D). As shown in Supplementary Figure S6E and F, the activity of HUVECs decreased when the concentration of HAP and Sr20-HAP was greater than 20 μg/mL. However, at low concentrations, especially at 5 μg/mL, Sr20-HAP significantly promoted the proliferation of HUVECs. HAP and Sr20-HAP had a greater effect on the viability of Raw264.7 cells at higher concentrations and could encourage the proliferation of Raw264.7 cells at 1 μg/mL. With the increase of HAP and Sr20-HAP concentrations and the extension of time, the viability of Raw264.7 cells gradually decreased (Supplementary Figure S6G and H).

**Figure 3.**
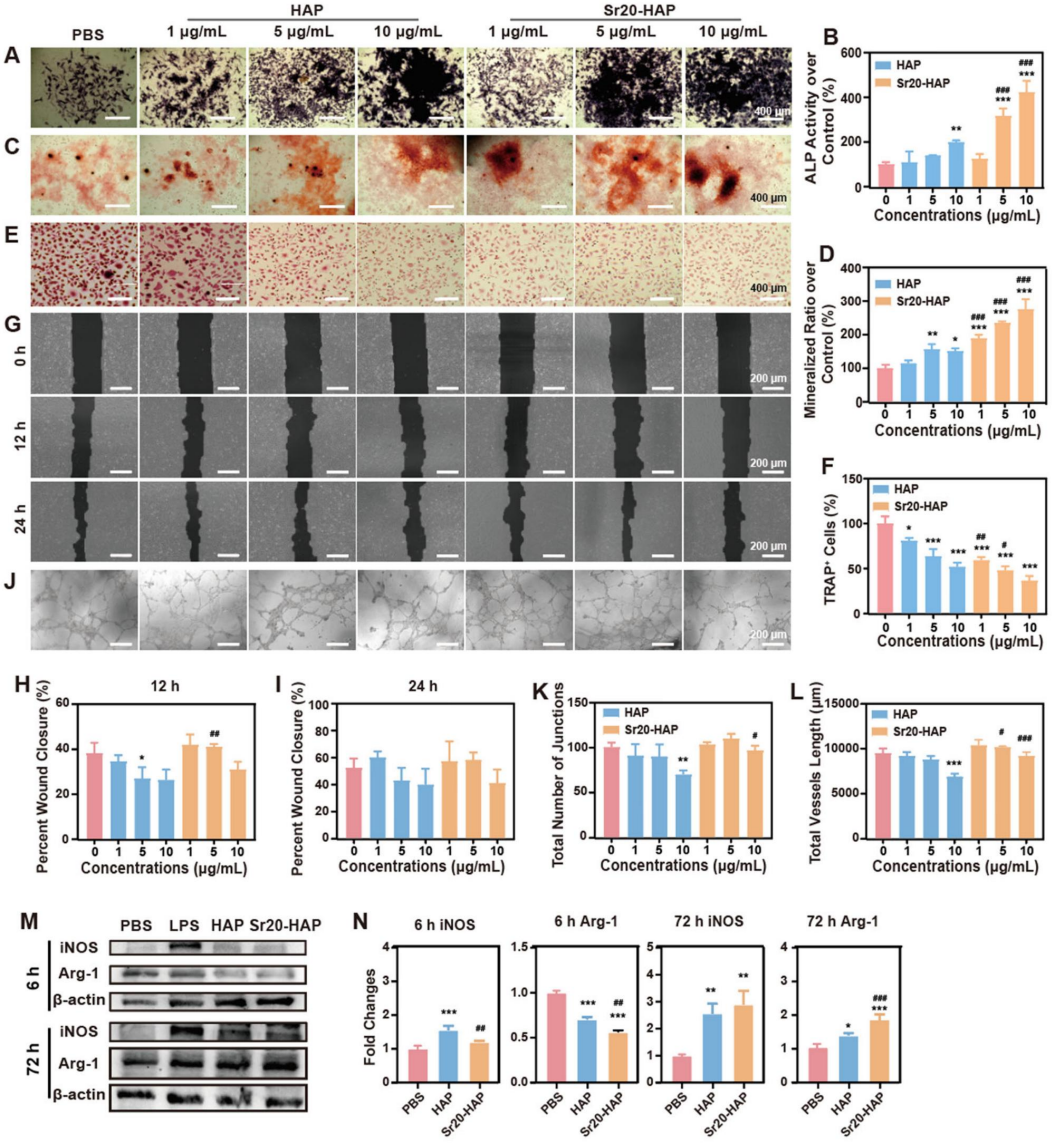
Cell viability and biological effects of HAP and Sr20-HAP for the bone microenvironment-related cells. BMSCs: (**A**) ALP staining images, (**B**) mineralization nudes staining by alizarin red, (**C**) quantitative results of ALP activity and (**D**) quantitative results of alizarin red staining. BMMNC: (**E**) TRAP staining images and (**F**) qualitative results of TRAP-positive cells with more than two nuclei. HUVECs: (**G**) Cell migration images in 0, 12 and 24 h, (**H**) quantitative results of cell migration for 12 h, (**I**) quantitative results of cell migration for 24 h, (**J**) vascularization images, (**K**) quantitative results of the angiogenesis crossing points, (**L**) quantitative results of the total length of vascularization. Raw264.7: (**M**) Expression of iNOS and Arg-1 in HAP and Sr20-HAP (1 μg/mL) cultured for 6 and 72 h, (**N**) The fold changes of iNOS and Arg-1 in M. Statistical analysis of treated groups versus 0 μg/ml group: * 0.01 ≤* P *< 0.05, ** 0.001 ≤* P *< 0.01 and *** *P *< 0.001. Statistical analysis of Sr20-HAP group versus HAP group at the same concentration: # 0.01 ≤* P *< 0.05, ## 0.001 ≤* P *< 0.01 and ### *P *< 0.001.

Sr ions can facilitate BMSCs’ osteogenic differentiation and mineralization by activating the Wnt/β-catenin signaling cascade and upregulating osteogenic transcription factors Runx2 and BMP-2 expression [45]. The impact of Sr20-HAP on osteogenic differentiation was evaluated via ALP and ARS assay. The expression of ALP in Sr20-HAP treated BMSCs was significantly enhanced with the increase of concentration, underscoring the promising potential of Sr20-HAP to boost osteoblast activity (Figure 3A). As shown in Figure 3B, BMSCs treated with Sr20-HAP show areas with higher calcification (red areas), a clear indication of enhanced osteogenic differentiation. According to quantitative data analysis, Sr20-HAP can significantly improve the osteogenic differentiation and mineralization ability of BMSCs (Figure 3C and D).

Meanwhile, Sr hinders the nuclear translocation of NFATc1, a key transcription factor for osteoclast differentiation, by downregulating RANKL expression or obstructing RANKL binding to its receptor RANK on osteoclast precursor cells, thereby reducing the formation of TRAP-positive osteoclasts [46–48]. Sr20-HAP showed inhibition effects on osteoclasts’ growth (Supplementary Figure S6C and D), which might further reduce osteoclasts’ activity, and thus, inhibit bone resorption. TRAP staining was employed to assess the impact of Sr20-HAP on the differentiation of bone marrow monocytes (macrophages) into osteoclasts. We first investigated whether Sr20-HAP can inhibit osteoclasts at the differentiation stage. As illustrated in Figure 3E and F, the majority of bone marrow monocytes (macrophages) transformed into osteoclasts (indicated by TRAP-positive red areas) when cultured in osteoclast induction media containing M-CSF and RANKL. In comparison to the control group, there was a notable decrease in the number of TRAP-positive multicellular osteoclasts in the Sr20-HAP treatment group, indicating that Sr20-HAP effectively inhibited osteoclast activity.

Bone is a highly vascularized tissue, and blood vessel proliferation influences bone formation [49, 50]. Sr ions can promote angiogenesis in bone tissue by inducing the secretion of vascular endothelial growth factor, supplying osteoblasts with nutrients and oxygen and thereby indirectly accelerating new bone formation [51]. The angiogenic capacity of Sr20-HAP was assessed by evaluating the migration and the network formation of HUVECs. Scratch test results showed that HAP and Sr20-HAP promoted HUVECs migration at 1 μg/mL (Figure 3G and H). The formation of reticular blood vessels can be seen in the angiogenesis experiment (Figure 3J). According to the quantitative data analysis of Image J, the Sr20-HAP group formed the most intersections of blood vessels and the longest total length of blood vessels (Figure 3K and L). These results underscore the potential of Sr20-HAP to significantly promote angiogenesis, offering hope for its role in bone formation.

Macrophages within bone tissue play a crucial role in preserving bone homeostasis [52, 53]. Bone disorders frequently occur alongside systemic or localized inflammation, where M1 macrophages (pro-inflammatory phenotype) and associated cytokines can stimulate osteoclast activity, enhance bone resorption and suppress bone formation. M2-type macrophages (anti-inflammatory phenotype) are important factors in promoting bone formation [54]. Sr ions are capable of suppressing the TLR4/MyD88/NF-κB pathway, thereby inhibiting the polarization of macrophages toward the M1 phenotype and reducing the expression of pro-inflammatory cytokines [55]. Additionally, Sr ions can activate the AMPK/mTOR pathway to induce the expression of genes related to the M2 macrophage phenotype [45]. To assess the potential inflammatory responses in the bone microenvironment, the M1 marker iNOS and M2 marker Arg-1 were detected in Raw264.7 cells after treatment with HAP and Sr20-HAP nanoparticles at various time points. As shown in Figure 3M, iNOS and Arg-1 showed a dynamic change with the increase of time. After 6 h of incubation, Arg-1 expression was absent in the HAP and Sr20-HAP groups. After incubation for 72 h, Arg-1 expression in the Sr20-HAP group was higher than that in the PBS and HAP groups. These results indicated that Sr20-HAP could significantly promote the polarization of Raw264.7 cells towards the M2 phenotype, thereby regulating the bone microenvironment and promoting bone recovery.

### Unveiling the impact of macrophage polarization on osteogenic differentiation and angiogenesis

**Figure 4.**
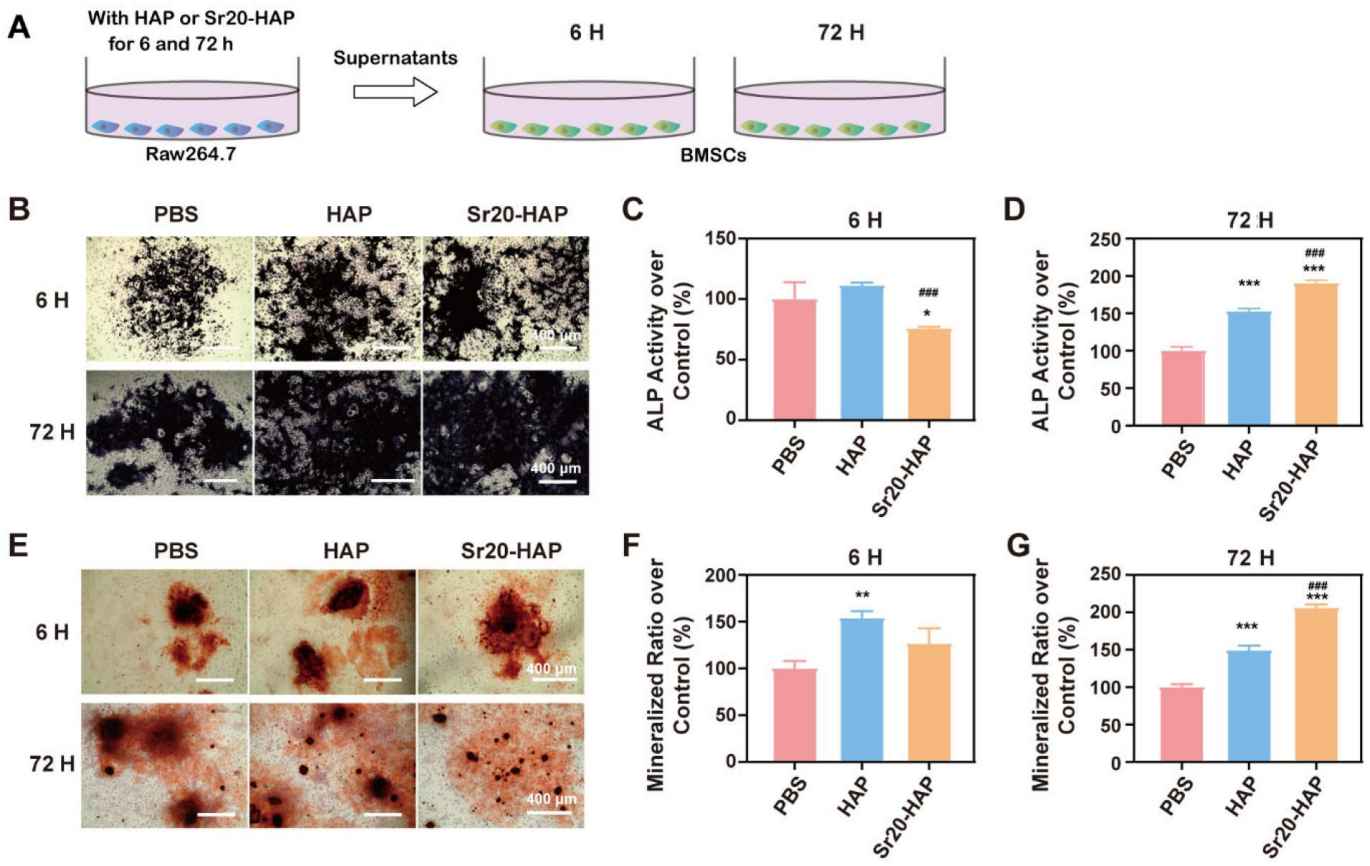
Osteogenic effect of BMSCs on the co-culture of Raw264.7 with HAP and Sr20-HAP for 6 and 72 h. (**A**) Illustration of the co-culture model on Raw264.7 and BMSCs; (**B**) ALP staining of BMSCs at 6H and 72H conditions; (**C**, **D**) Quantitative results of ALP activity of BMSCs; (**E**) Mineralization nudes staining of BMSCs at 6H and 72H conditions; (**F**, **G**) Quantitative results of alizarin red staining of BMSCs. Statistical analysis of treated groups versus 0 μg/mL group: * 0.01 ≤ *P *< 0.05, ** 0.001 ≤ *P *< 0.01 and *** *P *< 0.001. Statistical analysis of Sr20-HAP group versus HAP group: ### *P *< 0.001.

Macrophages of M2 type play an important role in promoting the proliferation and differentiation of mesenchymal cells and angiogenesis. Strontium-doped materials exhibited M2 anti-inflammatory properties during the bone repair phase [56]. Our investigation into the influence of macrophage phenotype on the osteogenic differentiation and the angiogenesis, particularly under HAP and Sr20-HAP treatment, has yielded significant results (Figure 4A). The osteogenic activity of BMSCs was significantly elevated compared to that observed in the single culture group, a result likely attributed to the anti-inflammatory and osteoblast factors secreted by M2 macrophages within the conditioned medium (Figure 4B and E). Quantitative analysis further confirmed the enhanced osteogenic activity of BMSCs under the influence of Sr20-HAP and conditioned media. Notably, the conditioned medium after Raw264.7 cultured with Sr20-HAP for 72 h (72H) showed a significant augmentation of these capacities (Figure 4C, D, F and G). These findings suggest that the synergistic effect induced by Sr20-HAP and M2 macrophages could be a promising avenue for activating osteogenic activity in the treatment of osteoporosis.

**Figure 5.**
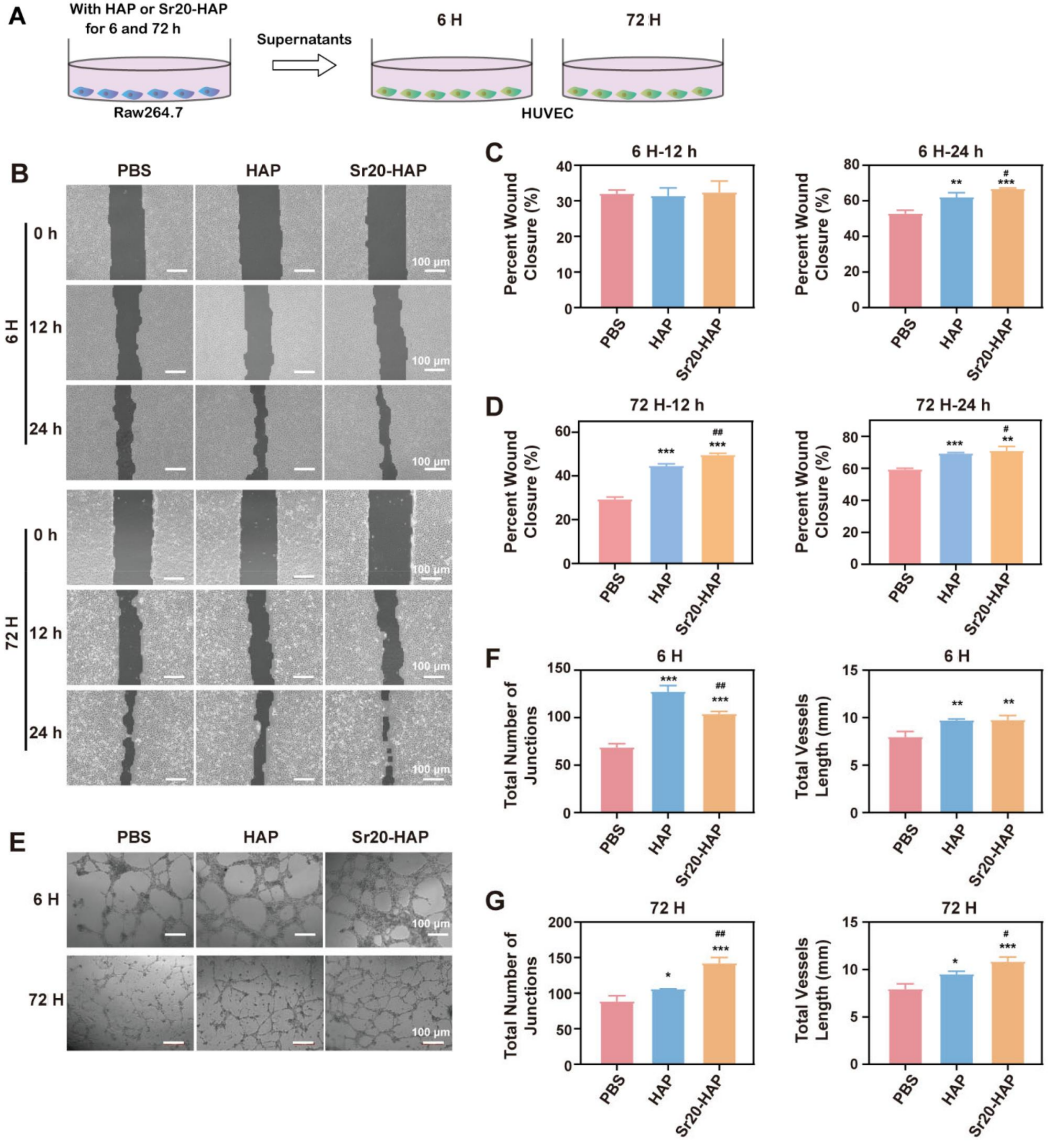
Vascularization effect of HUVECs on the co-culture of Raw264.7 with HAP and Sr20-HAP for 6 and 72 h. (**A**) Illustration of the co-culture model on Raw264.7 and HUVEC; (**B**) Cell migration images at 0, 12 and 24 h at 6H and 72H conditions, (**C**) Quantitative results of cell migration with 6 h-Raw264.7 (6H), (**D**) Quantitative results of cell migration with 72 h-Raw264.7 (72H), (**E**) Vascularization images at 6H and 72H conditions, (**F**) Quantitative results of the angiogenesis crossing points and the total length of vascularization with 6 h-Raw264.7 (6H), (**G**) Quantitative results of the angiogenesis crossing points and the total length of vascularization with 72 h-Raw264.7 (72H). Statistical analysis of treated groups versus 0 μg/mL group: * 0.01 ≤ *P *< 0.05, ** 0.001 ≤ *P *< 0.01 and *** *P *< 0.001. Statistical analysis of Sr20-HAP group versus HAP group: #0.01 ≤ *P *< 0.05 and ## 0.001 ≤ *P *< 0.01.

Our research has also shed light on the close relationship between bone formation and blood vessel formation, with the vascular invasion of bone injury hematoma being a crucial step in the healing process. After HUVECs were cultured in a conditioned medium (HUVECs medium and M2 medium, Figure 5A), their migration and angiogenesis capacity was found to be significantly higher than that of the single culture group (Figure 5B–G). The HAP and Sr20-HAP groups enhanced the migration and angiogenesis capacity of HUVECs. Still, the 72-h conditioned medium containing Sr20-HAP further significantly improved these capacities (Figure 5D and G), demonstrating the potential of the synergistic effect induced by Sr20-HAP and M2 macrophages in activating angiogenesis.

### Regulation of immune microenvironment and inhibition of osteoporotic bone loss in aged mice

**Figure 6.**
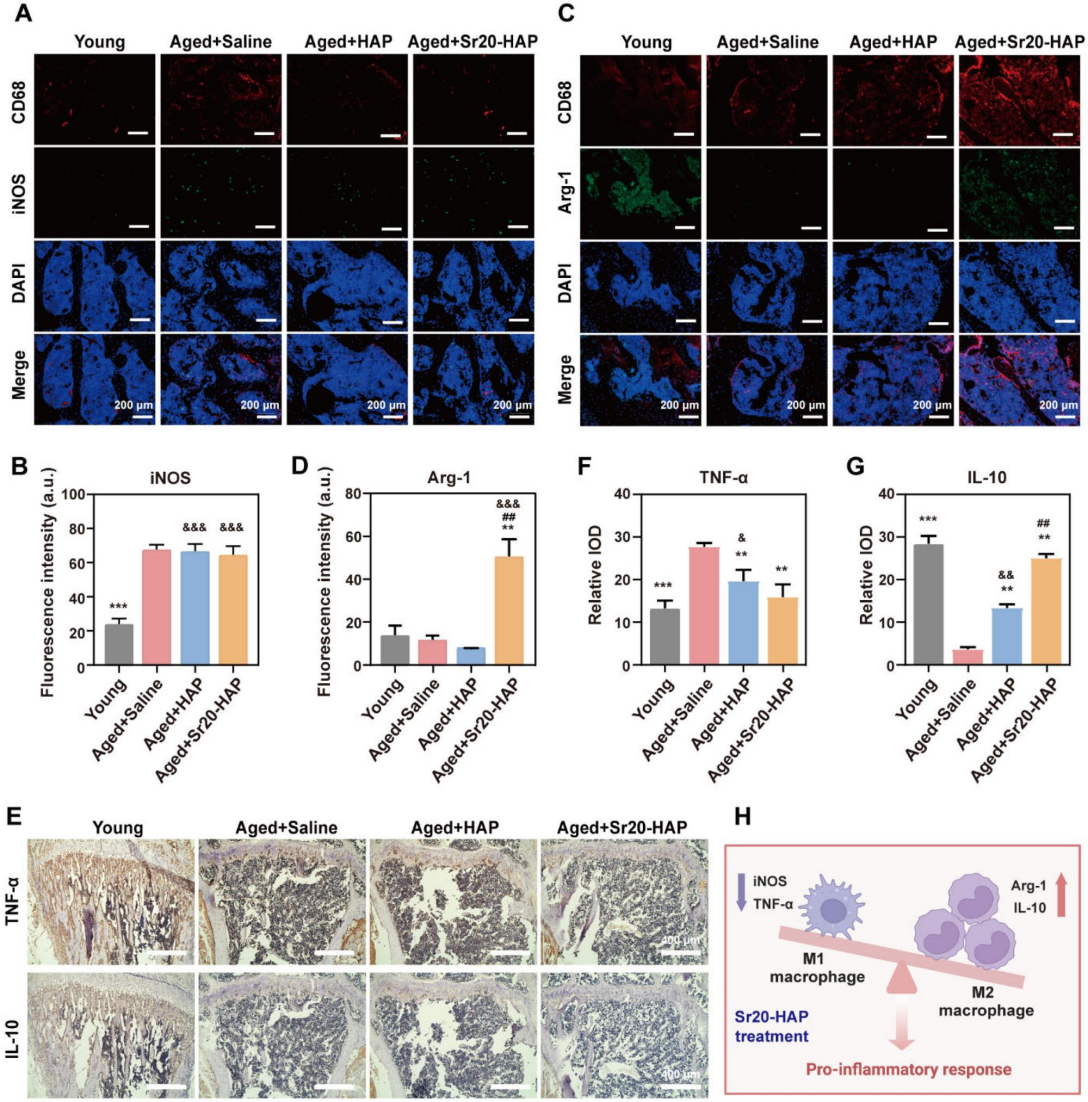
Regulation immune microenvironment in aged mice. (**A**) CD68 and Arg-1 immuno-fluorescence images of M2 macrophages in the bone slice. (**B**) CD68 and iNOS immuno-fluorescence images of M1 macrophages in the bone slice. (**C**) Quantitative results of Arg-1. (**D**) Quantitative results of iNOS. (**E**) Micrographs of tibias stained with IL-10 and TNF-α antibody. (**F**) Quantitative results of IL-10. (**G**) Quantitative results of TNF-α. (**H**) Illustration of macrophage type changes after Sr20-HAP treatment. Statistical analysis of young and treated groups versus Old-Saline group: ** 0.001 ≤ *P *< 0.01 and *** *P *< 0.001. Statistical analysis of Sr20-HAP group versus HAP group: ## 0.001 ≤ *P *< 0.01. Statistical analysis of HAP and Sr20-HAP groups versus young group: & 0.01 ≤ *P *< 0.05, && 0.001 ≤ *P *< 0.01 and &&& *P *< 0.001.

Macrophages are important members of the immune system, and their phenotypes and functions show remarkable plasticity. Bone injury healing is a complex dynamic process involving the interaction of multiple cellular and molecular signaling pathways, which can be divided into several stages: inflammation, regression and tissue recovery [57]. By regulating the phenotype of macrophages, the bone microenvironment can be improved to promote the formation and activity of osteoblasts, thereby increasing bone mass. Consequently, investigating the pathways and molecular mechanisms involved in the interaction between macrophages and bone cells is highly important for future clinical approaches to treating osteoporosis [44]. In histological analysis, immunohistochemistry and immunofluorescence staining of the tibia showed many iNOS-positive cells proliferating in aged mice administered with saline (Figure 6A and B). However, iNOS was significantly decreased after HAP treatment, and Arg-1 was increased considerably after Sr20-HAP treatment (Figure 6C and D). Meanwhile, pro-inflammatory TNF-α showed decrease in HAP and Sr20-HAP groups in aged mice (Figure 6E and F), and anti-inflammatory IL-10 showed increase in HAP and Sr20-HAP groups (Figure 6E and G). Moreover, the changes of TNF-α and IL-10 showed difference in HAP and Sr20-HAP groups (Figure 6F and G). The findings indicated that Sr20-HAP regulated the balance of macrophage phenotype and exerted an immunomodulatory effect by influencing the inflammatory microenvironment in mice with osteoporosis (Figure 6H).

**Figure 7.**
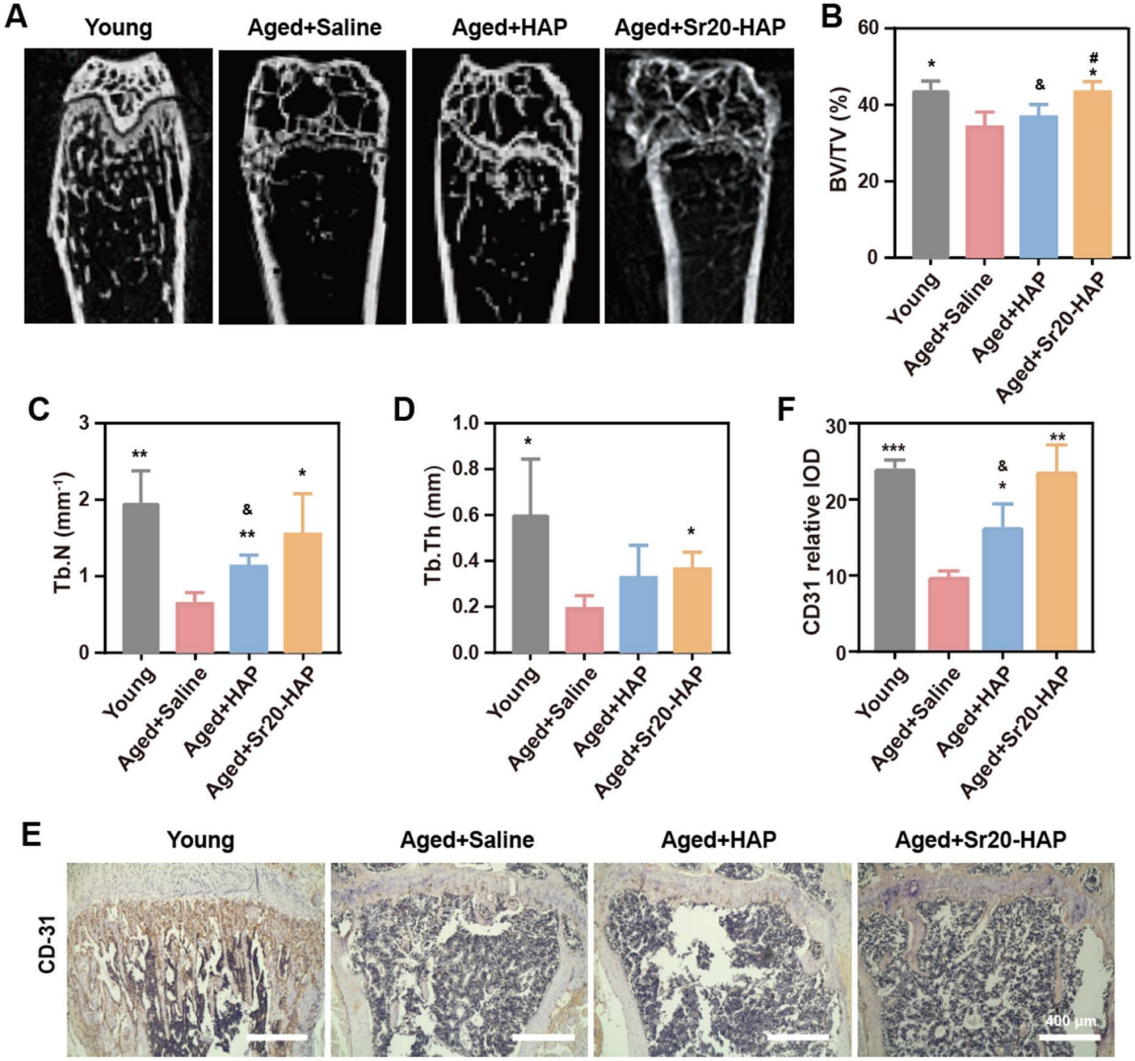
Promotion on bone formation in aged mice. (**A**) CT images of distal tibias. (**B**–**D**) Bone parameters of the tibias. (**E**) Immunohistochemical staining images of CD31. (**F**) Quantities results of CD31. Statistical analysis of young and treated groups versus Old-Saline group: * 0.01 ≤ *P *< 0.05, ** 0.001 ≤ *P *< 0.01 and *** *P *< 0.001. Statistical analysis of Sr20-HAP group versus HAP group: # 0.01 ≤ *P *< 0.05. Statistical analysis of HAP and Sr20-HAP groups versus young group: & 0.01 ≤ *P *< 0.05.

A naturally aged mouse model is more appropriate for assessing the effectiveness of immune cell-mediated bone healing in osteoporosis [34]. To further validate the reversing effect of Sr20-HAP on osteoporosis *in vivo*, female BALB/c mice at 12-month old were utilized as the osteoporosis model. Healthy mice aged 4 months were selected as controls based on BMD parameters. Each group of older mice received an intratibial injection of HAP or Sr20-HAP (12 mg/kg). Four weeks after the two samples were injected into the tibia, the changes of bone microstructure were analyzed by micro-computed tomography (micro-CT). CT images showed that Sr20-HAP could significantly improve bone repair in (Figure 7A). Bone-related parameters, tissue volume bone mass (BV/TV) and bone trabecular number (Tb. N) were considerably enhanced in the Sr20-HAP group, compared with those in the Saline and HAP groups (Figure 7B–D).

The formation of bone is closely related to the growth of blood vessels, and the establishment of effective vascularization in the early stage of bone microenvironment plays a crucial role in maintaining bone repair. In the stage of bone tissue repair, stem cells that form bone and blood vessel tissues will proliferate and differentiate to form cartilage callus. At the edge of the new cartilage tissue, periosteum will slowly expand, beginning the formation of bone. CD31 is the single best marker of endothelial differentiation. In this study, CD31 was used as an endothelial marker. Immunohistochemical staining was performed on the regulated bone microenvironment. Figure 7E and F showed that Sr20-HAP group could better express CD31 and promote the formation of blood vessels.

After HAP and Sr20-HAP were injected into the tibia of mice, H&E staining of major organs showed no significant changes in the histopathological morphology of the heart, liver, spleen, lung and kidney of mice (Supplementary Figure S7). Blood biochemical test results showed that the liver function (ALP, ALT, AST), renal function (CK, UA, Urea) and myocardial function (LDH, HBDH) of the mice were in the normal range, indicating that HAP and Sr20-HAP had good biosafety *in vivo* (Supplementary Figure S8).

## Conclusion

A variety of Srx-HAP nanocomposites with different Sr incorporating molar ratio concentrations (0%, 1%, 2%, 5%, 10%, 20%, 50%, 80% and 100%) were prepared by a facile hydrothermal route. The crystal phase, composition and morphology were characterized by XRD, SEM, TEM, ICP-OES and EDX elemental mapping images, respectively. The results demonstrated that the as-synthesized HAP and Srx-HAP samples showed monodisperse rod-like morphology and mesoporous structure. Among all the samples, Sr20-HAP showed the most predominant advantages in osteogenic differentiation and mineralization. Furthermore, the animal experiments demonstrated that the Sr20-HAP sample could evidently improve bone formation in the rat model of postmenopausal osteoporosis in comparison with the OVX group, and exhibited a similar bone turn-over with free ALN therapy group.

Subsequently, the effects of HAP and Sr20-HAP on various cell types within the bone microenvironment were investigated, including their individual impacts as well as intercellular interactions. Initially, when incubated separately with different cell types, it was observed that compared to HAP alone, a low concentration of Sr20-HAP significantly enhanced osteogenic differentiation and mineralization ability in BMSCs while inhibiting osteoclastogenesis. Moreover, it exhibited favorable biocompatibility towards endothelial cells (HUVECs) and notably augmented M2 polarization in macrophages (Raw264.7). Subsequently, conditioned media containing both materials were employed to interact with Raw264.7 for varying time periods while BMSCs and HUVECs were cultured accordingly. Remarkably, after 72 h of exposure to conditioned media containing Sr20-HAP, there was a significant improvement in osteogenic differentiation and mineralization capacity of BMSCs along with enhanced migration and angiogenesis in HUVECs.

Meanwhile, the regulatory effect of Sr20-HAP on the bone microenvironment *in vivo* was investigated using a 12-week-old aged mouse model of bone injury. After intratibial injection of HAP and Sr20-HAP for 4 weeks, compared to the young group at 4 weeks old, the older mice in the saline group exhibited a significant upregulation in M1 macrophage marker iNOS expression and negligible expression of M2 macrophage marker Arg-1 in bone tissue slices. The pro-inflammatory cytokine TNF-α and anti-inflammatory cytokine IL-10 were significantly downregulated, indicating an inflammatory microenvironment in aged mouse bone tissue with polarization towards M1 macrophages. In contrast, there was no substantial difference observed in iNOS expression levels between the Sr20-HAP group and aged mice. However, Arg-1 expression levels were significantly increased while TNF-α expression was reduced, suggesting that Sr20-HAP can effectively regulate macrophage polarization towards M2 phenotype *in vivo*. CT results demonstrated effective promotion of bone repair by Sr20-HAP. Blood biochemistry analysis and H&E staining results confirmed excellent biocompatibility of these nanomaterials *in vivo*.

The present study investigates the mechanism of action of Sr-HAP in regulating bone formation at both cellular and animal levels, providing a theoretical basis and experimental evidence for the application of Sr-HAP in bone tissue repair. This study offers novel insights into the treatment of bone injuries and contributes to the development of innovative approaches for bone repair therapy.

## Supplementary Material

rbaf066_Supplementary_Data
