# Supplementary material for: Strontium-incorporated hydroxyapatite nanocomposites promoting bone formation and angiogenesis by modulating M2 macrophage polarization in the bone microenvironment
Source: Regen Biomater. 2025 Jun 23;12:rbaf066. doi: 10.1093/rb/rbaf066 (PMC12341688; doi:10.1093/rb/rbaf066)
Supplement: rbaf066_Supplementary_Data [file rbaf066_supplementary_data.docx]

Supplementary Information

**Strontium-incorporated hydroxyapatite nanocomposites promoting bone formation and angiogenesis by modulating M2 macrophage polarization in the bone microenvironment**

Jing Li ^1,†^, Cuimiao Zhang ^1,†^, Jiayi Li ^1^, Ruijing Gao ^1^, Mengzhen Yang ^1^, Linkang Yu ^1^, Wei Zhang ^3^, Guoqiang Zhou ^1,2^, Wenzeng Shen ^2^, Jinchao Zhang ^1,^*, Guang Jia ^1,^*, Kun Ge ^1,^*

^1^ College of Chemistry & Materials Science, Key Laboratory of Medicinal Chemistry and Molecular Diagnosis of Ministry of Education, State Key Laboratory of New Pharmaceutical Preparations and Excipients, Chemical Biology Key Laboratory of Hebei Province, Hebei University, Baoding, 071002, China

^2^ College of Basic Medical Science, Hebei University, Baoding, 071000, China

^3^ Institute of Biomedical and Health Engineering, Shenzhen Institute of Advanced Technology Chinese Academy of Sciences, Shenzhen, 518055, China

^#^ The authors contributed equally to this work.

*Corresponding address. E-mail: [jczhang6970@163.com](mailto:jczhang6970@163.com) (J. Zhang), [guangjia2001@163.com](mailto:guangjia2001@163.com) (G. Jia), [gekun@hbu.edu.cn](mailto:gekun@hbu.edu.cn) (K. Ge)

**Preparation of pure HAP nanoparticles**

In brief, 3 mmol of Ca(NO_3_)_2_, 0.5 g of CTAB, and 10 mL of ammonia solution (NH_3_•H_2_O) were dissolved in deionized water to form 40 mL of solution Ⅰ. Then, 6 mmol of trisodium citrate and 2 mmol of (NH_4_)_2_HPO_4_ were added into 20 mL of H_2_O to form solution Ⅱ. After vigorous agitation for 30 minutes, solution Ⅱ was introduced into solution Ⅰ. After additional stirring for 20 minutes, the as-obtained mixing solution was transferred into a Teﬂon bottle (100 mL) held in a stainless-steel autoclave, sealed, and maintained at 180°C for 24 hours. As the autoclave cooled to room temperature naturally, the precipitate was separated by centrifugation and washed with deionized water and ethanol in sequence. Then, the as-obtained product was redispersed in 150 mL of acetone and reﬂuxed at 80°C for 48 hours to remove the residual template CTAB. Finally, the precipitate was separated by centrifugation and dried in a vacuum at 70°C for 24 hours to obtain the pure HAP sample.

**Characterizations**

The X-ray diffraction (XRD) patterns of the samples were characterized on a D8 Advance diffractometer (Bruker) using Cu Ka radiation in the range of 20~80° in steps of 0.02° per second for the determination of crystal structure and phase. The morphology and composition of the samples were observed using a scanning electron microscope (SEM) (JEOL, JSM-7500F, Japan) and transmission electron microscopy (TEM) from an FEI Tecnai G2 S-TWIN. Nitrogen adsorption/desorption analysis was conducted using a Micromeritics ASAP 2020 M apparatus. The surface areas of sample powders were calculated according to the Barrett-Emmett-Teller (BET) equation using the data between 0.05 and 0.35. The relative pressure P/Po of the isotherm was studied between 0.01 and 1.0.

**MC3T3-E1 cell viability and osteogenic differentiation**

Pre-osteoblasts cell line MC3T3-E1 was employed for cell safety and osteogenic differentiation of Srx-HAP. MC3T3-E1 cells were cultured in α-MEM with 10% FBS and 100 units/mL penicillin-streptomycin. The osteogenic induction medium (OS) was supplemented with 10^-8^ mol/L dexamethasone, 10 mM β-glycerophosphate, and 50 μg/mL ascorbic acid. All the incubations were in humidiﬁed atmospheres of 5% CO_2_ at 37°C and the medium changed every two days.

First, the cell viability was assessed with an MTT assay. In Brief, the MC3T3-E1 cells were seeded into 96-well plates at a density of 2×10^3^ cells/well. After overnight culture, different concentrations of Srx-HAP nanocomposites (0, 1, 5, 10, 20, 40, and 80 μg/mL) were incubated with cells for 24, 48, and 72 hours. At predetermined time points, MTT solutions were added and OD_570_ values were tested by a microplate reader (M4 SpectraMax, Molecular Devices, USA).

The osteogenic differentiation of MC3T3-E1 cells was evaluated by determining ALP activity and mineralization. MC3T3-E1 cells were seeded into 24-well plates at a density of 2×10^4^ cells/well. After incubation with 40 μg/mL Srx-HAP for 14 days, the ALP activity was performed by ALP detection kit based on the manufacturer's instructions and normalized to the total protein amount detected by a BCA assay kit. After being cultured with Srx-HAP for 20 days, cells were fixed in 95% ethanol and stained with 0.1% Alizarin Red S. The mineralized nodules in each group were captured by a microscope (OLYMPUS, Japanese) and quantified by 10% cetylpyridinium chloride solution.

**Ovariectomy osteoporotic rat experiments**

Ovariectomy was performed in 12-week-old female Wistar rats with proper anesthesia using pentobarbital sodium [100 mg/kg body weight, intraperitoneally (i.p.)] to establish the estrogen depletion animal model]. Twelve weeks post OVX, rats were divided into ten groups (6 rats/group) and administered with 100 μg/kg different treatments once intravenously: the saline group (OVX), free alendronate group (ALN), and four Srx-HAP groups (HAP, Sr10-HAP, Sr20-HAP, and Sr100-HAP). The sham group (Sham) was administered with saline as a negative control. Another 12 weeks later, rats were weighed and anesthetized. Blood was collected from the aorta abdominals and serum was harvested. A serum bone resorption marker CTX-1 was measured with a rat CTX-1 enzyme immunoassay kit. In addition, rats’ hearts, livers, spleens, lungs, kidneys, and femoral bones were extracted. The femoral bones were cleared of connective tissues and immersed in tissue fixative for further analysis. Other tissues were stored in formalin solutions and then made paraffin-embedded blocks for hematoxylin and eosin (H&E) staining.

**Bone microstructure analysis**

The trabecular structures of the femora distal region in the different groups were characterized by micro-CT (SkyScan 1172, Bruker). Bone morphometric parameters were measured, such as bone mineral density (BMD), bone surface/bone volume ratio (BS/TV), bone volume/tissue volume ratio (BV/TV), trabecular number (Tb.N.), and trabecular thickness (Tb.Tn.). Then, the femurs were decalcified with 10% EDTA and prepared for H&E and Masson stains for histological observation.

**Cell viability of BMSCs, BMMNCs, HUVECs, and Raw264.7 cells on HAP and Sr20-HAP**

We assessed the cell viability of bone marrow-derived mesenchymal stem cells (BMSCs), bone marrow mononuclear cells (BMMNCs), human umbilical vein endothelial cells (HUVECs), and Raw264.7 cells on HAP and Sr20-HAP was assessed in this study, considering their significance in the bone microenvironment. The cells were plated at a density of 2×10^3^ cells/well in 96-well plates and treated with complete media containing varying concentrations of HAP or Sr20-HAP at a concentration of 0, 1, 5, 10, 20, 40, and 80 μg/mL, followed by incubation for either 24 or 48 hours. The CCK-8 assay was utilized to evaluate cell viability following the manufacturer's protocol.

**Osteogenic and osteoclast differentiation on HAP and Sr20-HAP**

For alkaline phosphatase (ALP) staining, ALP activity, and mineralization analysis, BMSCs were seeded in 48-well plates at 5×10^6^ cells/well density and cultured for 6 days. HAP and Sr20-HAP with concentrations of 0, 1, 5, and 10 μg/mL were added to the culture. After 7 days of culture, BMSCs were stained using an ALP staining kit, and images were captured using a microscope (EVOS FL Auto Cell Imaging System, ThermoFisher Scientific). Following the instructions, BMSCs were quantified using an ALP quantitative kit. After culturing for 14 days, BMSCs were stained with an alizarin red solution, and a microscope took the images. The stained mineralized nodules were quantified using a commonly used method involving a 10% cepirichloramine solution and measured by a microplate reader at OD570 nm.

The BMMNCs were seeded in 24-well plates at a density of 5×10^6^ cells per well and cultured in DMEM medium supplemented with 30 ng/mL M-CSF for 3 days. Subsequently, the BMMNCs were maintained in a DMEM medium containing 30 ng/mL M-CSF and 50 ng/mL RANKL while treated with HAP and Sr20-HAP at 0, 1, 5, and 10 μg/mL concentrations. After 14 days, the BMMNCs were subjected to tartrate resistant acid phosphatase (TRAP) staining using appropriate kits. The positively stained TRAP cells were visualized and quantified under a microscope.

**Vascularization of HUVECs on HAP and Sr20-HAP**

HUVECs were seeded in 6-well plates at a density of 2×10^5^ cells/well and cultured in a serum-free medium for 24 hours to induce starvation. Subsequently, a linear wound was created using a 200 μL tip, followed by treatment with HAP and Sr20-HAP media at final concentrations of 0, 1, 5, and 10 μg/mL. A microscope captured the wound locations after 0, 12, and 24 hours, and cell mobility was quantified using ImageJ.

Before experimentation, HUVECs were cultured in a medium containing only 1% serum for 24 hours. Then, HUVECs were seeded onto matrigel-coated (at a volume ratio 1:1) plates at a density of approximately 1×10^5^ cells/well. After treating the cells with varying concentrations (0, 1, 5, or 10 μg/mL) of HAP and Sr20-HAP for an incubation period of 8 hours, total vessel length and intersections were captured by a microscope and analyzed by ImageJ.

**Macrophage phenotype of Raw264.7 cells on HAP and Sr20-HAP**

The Raw264.7 cells were seeded in 6-well plates at a density of 2×10^5^ cells/well and treated with HAP and Sr20-HAP at 0 and 1 μg/mL concentrations for 6, 24, 48, and 72 hours. Subsequently, western blotting analyzed the extracted proteins from the Raw264.7 cells subjected to different treatments for polarization phenotypic markers.


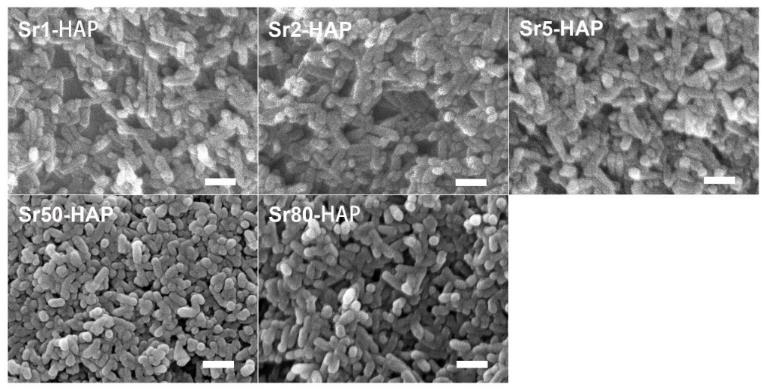


**Figure S1.** SEM images of Sr1-HAP, Sr2-HAP, Sr5-HAP, Sr50-HAP, and Sr80-HAP. Scale bar is 100 nm.


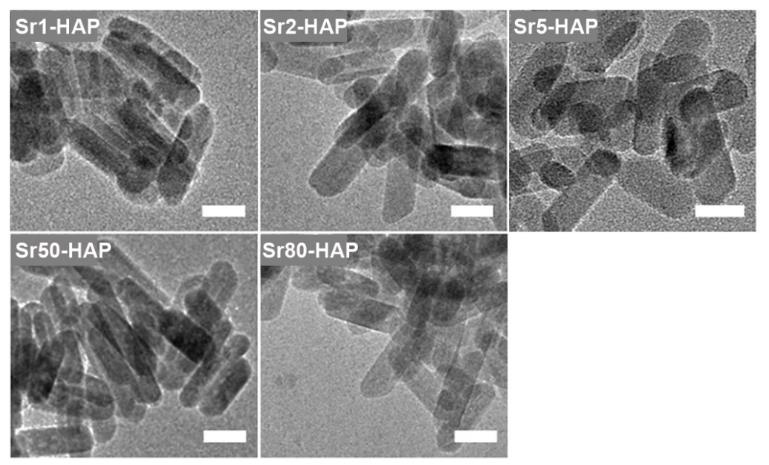


**Figure S2.** TEM images of Sr1-HAP, Sr2-HAP, Sr5-HAP, Sr50-HAP, and Sr80-HAP. Scale bar is 25 nm.


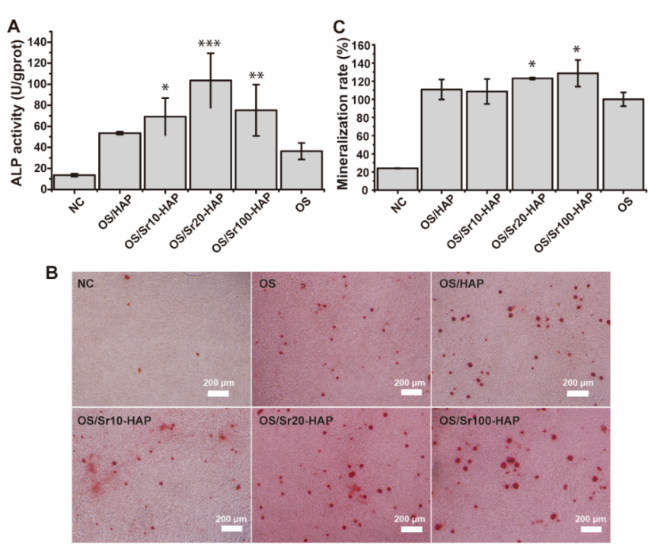


**Figure S3.** Osteogenic differentiation and mineralization of MC3T3-E1 after being treated with HAP and Srx-HAP samples. (A) ALP activity, (B) Alizarin red S staining images, (C) Mineralization rates of semiquantiﬁcational alizarin red S-based assays carried out by OS group. **p*<0.05, ***p*<0.01, and ****p*<0.001 for the different concentrations of Srx-HAP groups *vs* OS group.


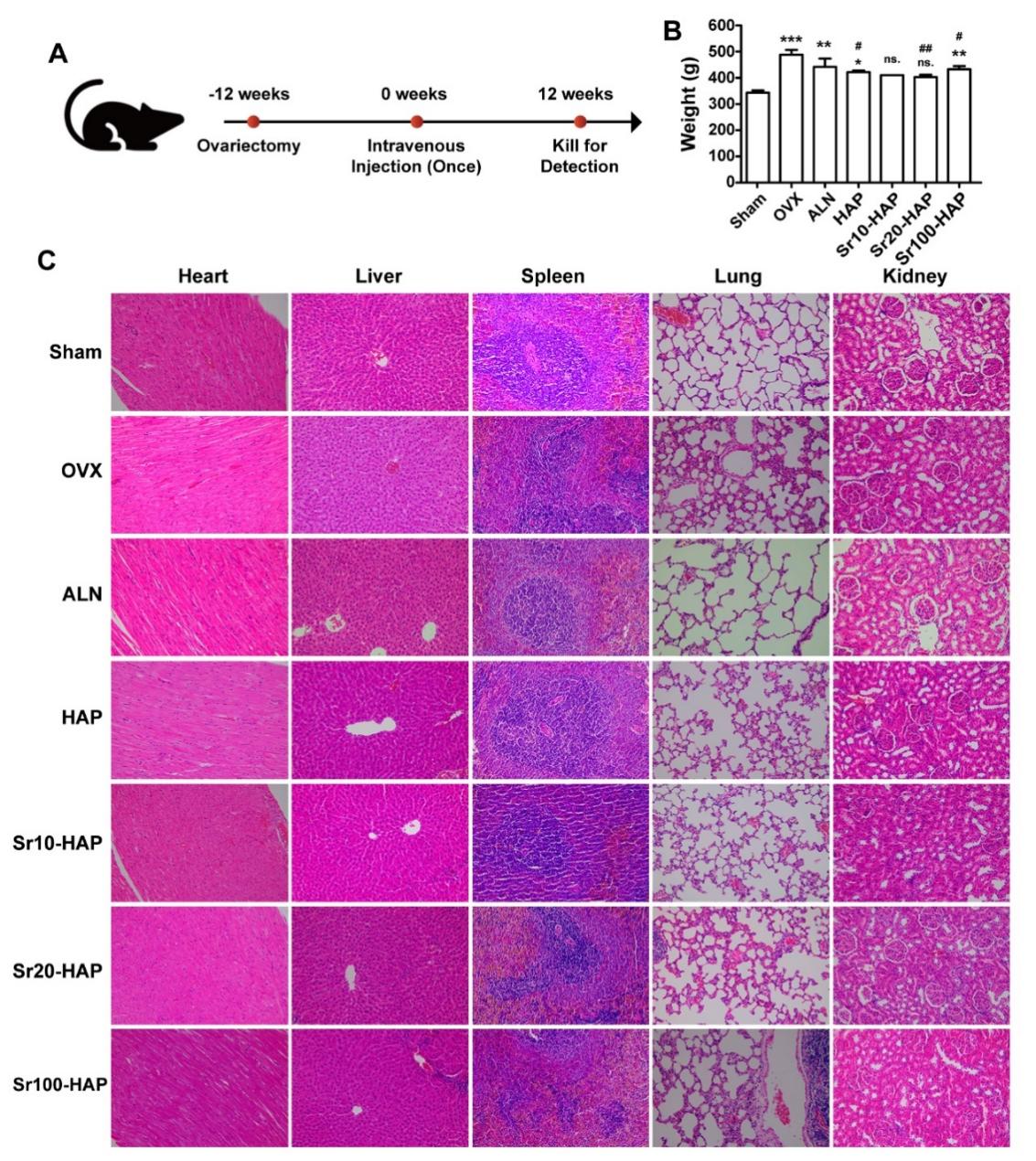


**Figure S4.** Biosafety evaluation of HAP and Srx-HAP samples on osteoporosis rats. (A) Timeline of the treatment schedule. (B) Rats’ bodyweights. (C) H&E images of main organ slices. **p*<0.05, ***p*<0.01, and ****p*<0.001 for different groups *vs* Sham group. ^#^*p*<0.05 and ^##^*p*<0.01 for different groups *vs* OVX group.


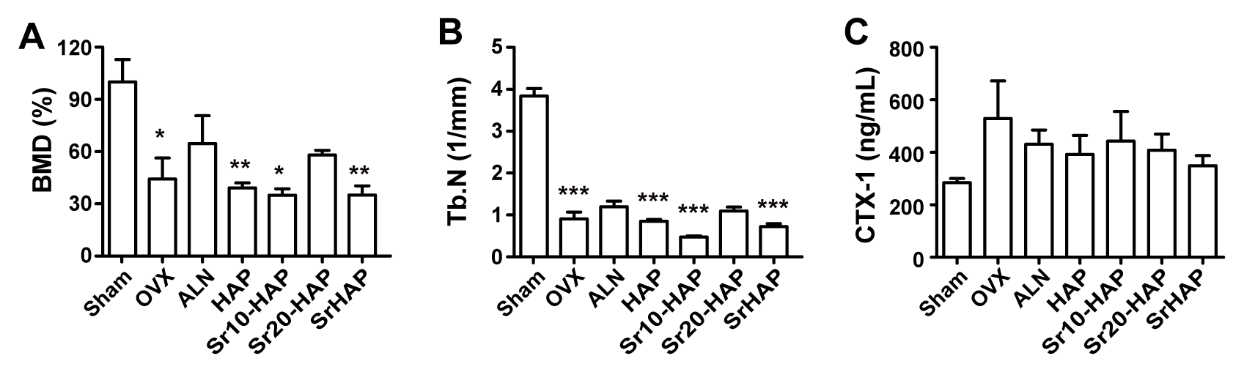


**Figure S5.** Osteolysis inhibition of distal femur after being treated with Srx-HAP. (A and B) Bone parameters: BMD (A) and Tb.N (B) from Figure 2B. (C) Serum CTX-1 content detected by ELISA.


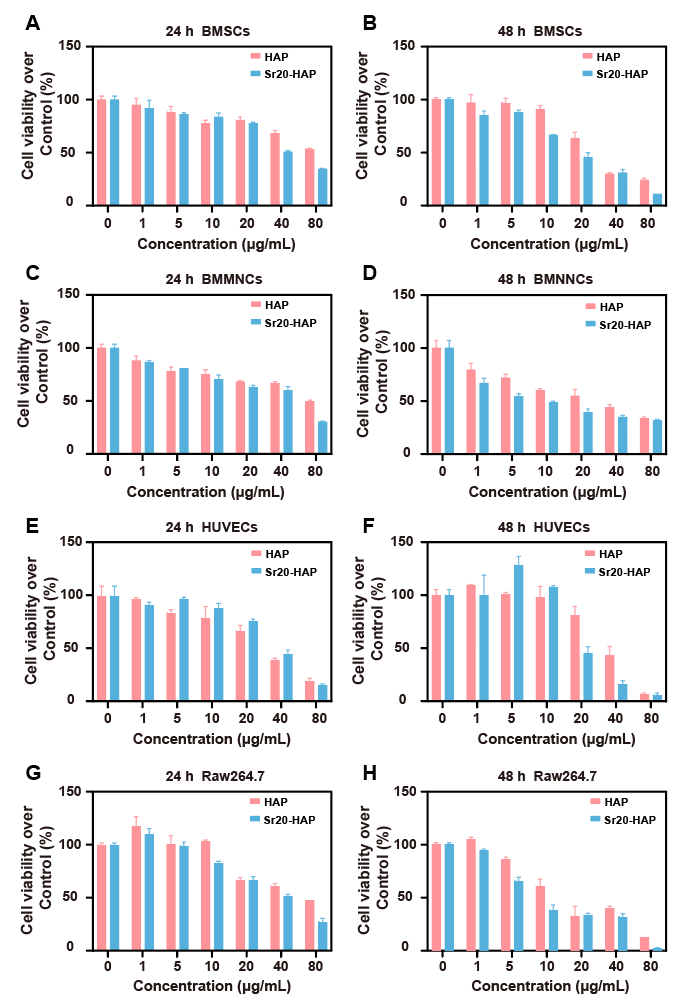


**Figure S6.** Cell viabilities of BMSCs (A and B), BMMNCs (C and D), HUVECs (E and F), and Raw264.7 (G and H) after incubation with the HAP and Sr20-HAP samples at different concentrations at 24 and 48 hour.


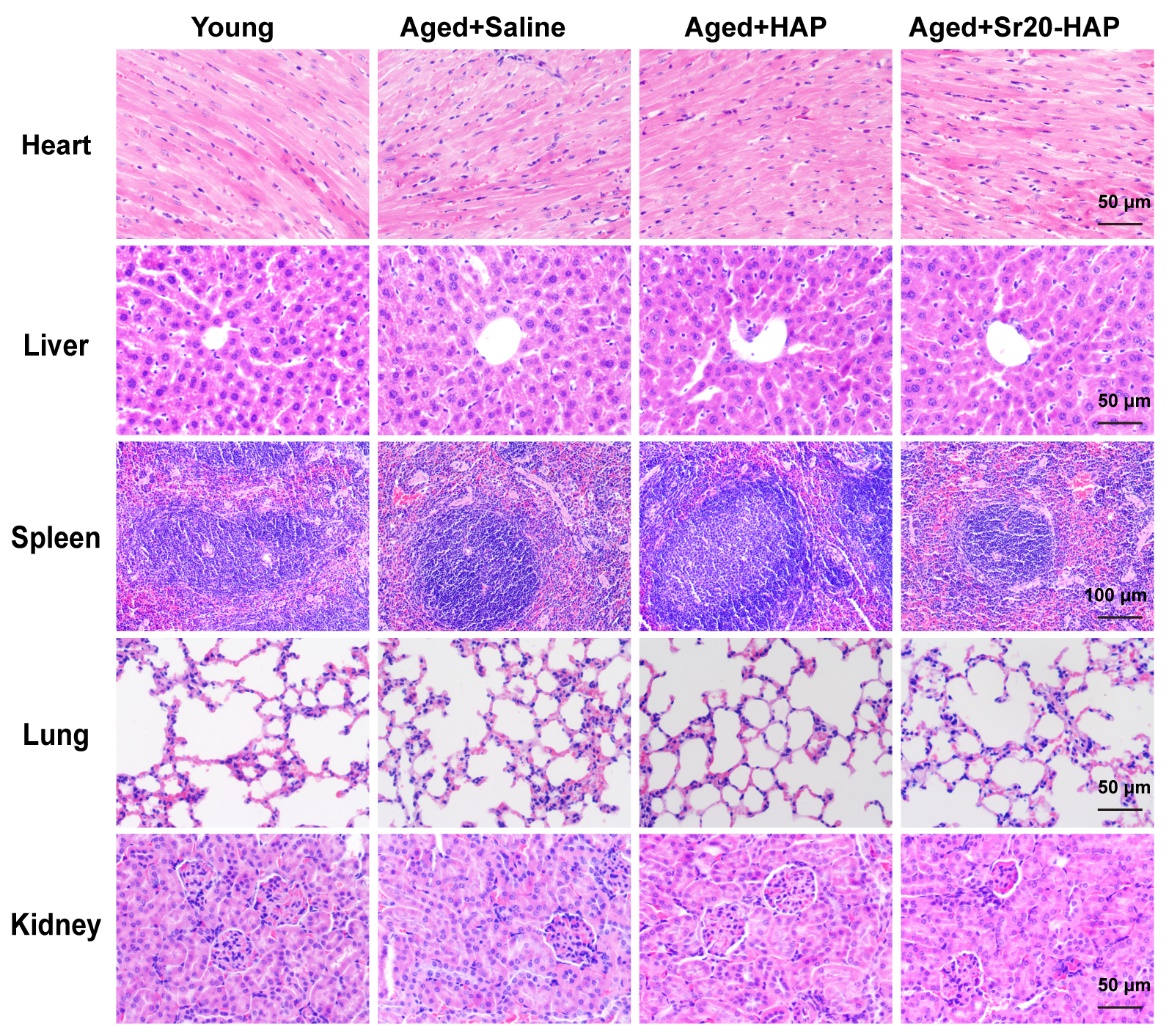


**Figure S7.** H&E staining of heart, liver, spleen, lung and kidney after the treatment of HAP and Sr20-HAP in aged mice.

**
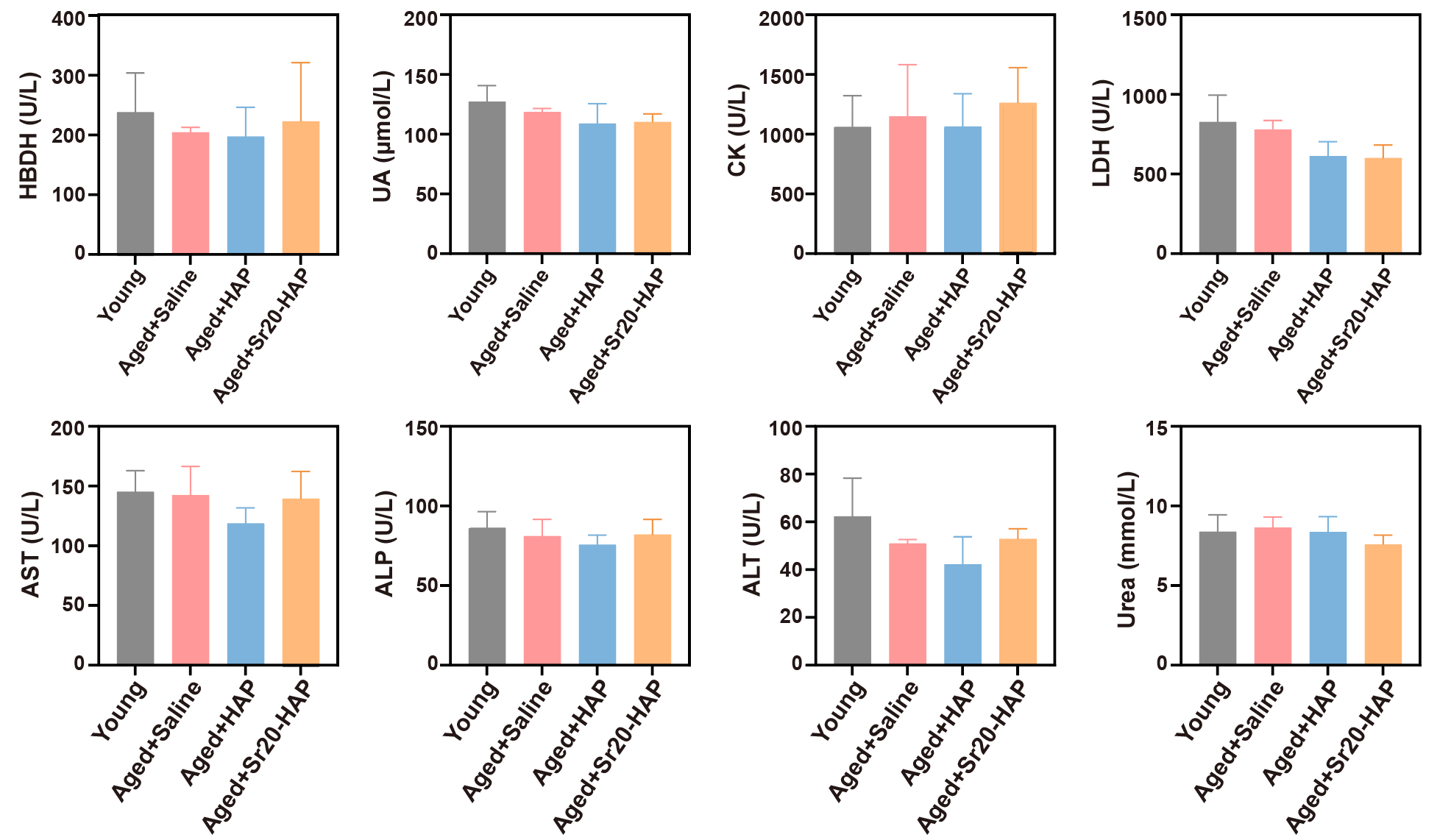
Figure S8.** Blood biochemical data after the treatment of HAP and Sr20-HAP in aged mice.

**Table S1.** Strontium ratios for Srx-HAP samples detected by ICP-OES

| Sample Name | Sr1-HAP | | Sr2-HAP | Sr5-HAP | Sr10-HAP | Sr20-HAP | Sr50-HAP | Sr80-HAP | Sr100-HAP |
| --- | --- | --- | --- | --- | --- | --- | --- | --- | --- |
| Synthesis-Sr feeding ratio (%) | 1 | 2 | | 5 | 10 | 20 | 50 | 80 | 100 |
| Analysis-Sr Molar Percentage (%) | 1.225 | 2.353 | | 6.264 | 12.339 | 22.797 | 54.277 | 80.443 | 99.566 |

**Table S2.** Zeta potential, BET surface area, and pore volume of HAP and Srx-HAP

| Sample Name | HAP | Sr10-HAP | Sr20-HAP | Sr100-HAP |
| --- | --- | --- | --- | --- |
| Zeta potential (mV ±1SD) | -5.41±0.25 | -5.60±0.19 | -7.44±0.24 | -8.47±0.3 |
| BET surface area (m^2^/g) | 60.38 | 52.66 | 40.88 | 29.61 |
| Pore volume(cm^3^/g) | 0.31 | 0.27 | 0.23 | 0.18 |

**Table S3.** Biochemical parameters in the serum of rats after intraperitoneal injection with Srx-HAP samples with/without ALN

| Groups | Urea  (mmol/L) | Cr  (umol/L) | UA  (umol/L) | CK  (U/L) | HBDH  (U/L) | LDH  (U/L) | ALP  (U/L) | AST  (U/L) | ALT  (U/L) | AST/ALT |
| --- | --- | --- | --- | --- | --- | --- | --- | --- | --- | --- |
| Sham | 8.46±1.52 | 51.25±9.00 | 118.25±41.21 | 2709.00±1985.81 | 920.25±319.91 | 3545.00±1401.39 | 45.50±27.11 | 342.50±133.43 | 58.75±29.41 | 6.1±0.92 |
| OVX | 7.49±0.07 | 73.33±10.26 | 187.00±73.53 | 3553.33±1568.90 | 946.33±199.76 | 3971.00±824.00 | 58.33±12.50 | 290.00±26.00 | 50.00±9.64 | 5.9±0.59 |
| ALN | 7.86±1.08 | 61.33±16.04 | 93.67±2.08 | 5633.67±2582.72 | 948.67±145.21 | 3983.67±446.13 | 64.33±27.06 | 392.67±154.87 | 139.33±85.80 | 3.5±1.58* |
| HAP | 7.67±1.03 | 60.33±7.23 | 276.00±41.15** | 2468.00±1030.01 | 1029.00±196.30 | 4473.00±712.55 | 41.67±4.04 | 312.67±29.19 | 70.33±22.81 | 4.7±1.1 |
| Sr10-HAP | 7.14±1.53 | 62.50±9.19 | 220.00±21.21 | 2422.50±446.18 | 843.00±18.38 | 3739.50±294.86 | 52.00±11.31 | 246.00±41.01 | 53.50±13.44 | 4.6±0.4 |
| Sr20-HAP | 7.08±0.57 | 63.25±6.99 | 156.00±36.74 | 1993.50±646.43 | 649.50±180.36 | 2899.50±974.34 | 42.50±11.82 | 210.25±52.21 | 42.00±10.10 | 5.0±0.85 |
| Sr100-HAP | 8.34±0.47 | 65.75±5.50 | 145.75±46.02 | 1115.25±230.25 | 727.75±97.05 | 3214.25±535.30 | 36.75±9.36 | 198.25±14.52 | 44.50±7.14 | 4.6±1.05 |

**Table S4.** Blood-element test of rats after intraperitoneal injection with Srx-HAP samples

| Groups | WBC | RBC | HGB | HCT | MCV | MCHC | PLT | RDW | |
| --- | --- | --- | --- | --- | --- | --- | --- | --- | --- |
|  |  |  |  |  |  |  |  | CV | SD |
| Sham | 4.17±1.25 | 9.44±1.21 | 178.00±21.34 | 55.06±6.82 | 58.34±1.25 | 323.60±7.80 | 724.80±154.84 | 15.86±2.12 | 23.44±0.73 |
| OVX | 4.12±0.24 | 9.21±0.73 | 179.00±2.83 | 53.10±4.67 | 57.35±0.92 | 319.50±2.12 | 790.50±68.59 | 16.50±0.42 | 23.00±0.71 |
| ALN | 3.60±2.17 | 9.31±0.31 | 178.50±4.95 | 54.65±1.91 | 58.70±0.14 | 326.50±2.12 | 823.00±149.91 | 16.35±0.21 | 23.80±0.42 |
| HAP | 5.69±0.77 | 9.38±0.59 | 168.33±11.02 | 52.33±3.18 | 55.83±0.15 | 321.67±2.31 | 907.33±183.30 | 16.87±1.59 | 24.13±2.41 |
| Sr10-HAP | 6.06±1.37 | 8.98±0.83 | 167.00±7.07 | 52.25±2.62 | 58.35±2.47 | 319.50±2.12 | 976.50±125.16 | 16.20±0.14 | 24.80±2.55 |
| Sr20-HAP | 4.11±0.46 | 8.83±0.57 | 168.75±6.13 | 52.10±1.62 | 59.08±2.08 | 323.50±2.08 | 752.75±28.12 | 14.40±0.62 | 23.78±3.37 |
| Sr100-HAP | 9.16±2.92 | 8.25±0.36 | 158.00±4.24 | 48.08±1.63 | 58.28±2.19 | 328.75±6.13 | 740.50±102.09 | 14.50±1.47 | 24.23±2.24 |
